# Technetium-99 determination in low-volume samples from the global environment with accelerator mass spectrometry

**DOI:** 10.1039/d6va00067c

**Published:** 2026-06-05

**Authors:** Karin Hain, Stephanie Adler, Thomas Faestermann, L. Keith Fifield, Michaela B. Froehlich, Fadime Gülce, Dominik Koll, Gunther Korschinek, Martin Martschini, Francesca Quinto, Stefan Pavetich, Johanna Pitters, Georg Rugel, Stephen Tims, Jan Welch, Andreas Wiederin, Masatoshi Yamada

**Affiliations:** a University of Vienna, Faculty of Physics Währinger Str. 17 1090 Vienna Austria karin.hain@univie.ac.at; b Technische Universität München, Physik Department James-Franck-Straße 1 Garching D-85748 Germany; c The Australian National University, Department of Nuclear Physics and Accelerator Applications Canberra ACT 2601 Australia; d Karlsruhe Institute of Technology (KIT), Institute for Nuclear Waste Disposal (INE) Hermann-von-Helmholtz Platz 1 76344 Eggenstein-Leopoldshafen Germany; e Helmholtz-Zentrum Dresden-Rossendorf Bautzner Landstraße 400 01328 Dresden Germany; f TRIGA Center Atominstitut, TU Wien Stadionallee 2 1020 Vienna Austria; g Marine Ecology Research Institute 300 Iwawada, Onjuku Chiba 299-5105 Japan

## Abstract

The long-lived fission product technetium-99 (^99^Tc, *t*_1/2_ = (2.111 ± 0.012) × 10^5^ years) was successfully detected in small-volume (10 L, and 1 g, respectively) environmental samples such as Pacific Ocean and river water, Antarctic snow, and peat. Its presence in the environment is almost exclusively of anthropogenic origin and the determined levels are attributed to nuclear weapons testing in the 1950s and 60s as no local contamination source is known to be present at these sampling sites. A unique capability for Accelerator Mass Spectrometry (AMS) measurements of environmental ^99^Tc at unprecedented sensitivity was established, first using the Gas-filled Analysing Magnet System (GAMS, Germany) and after its shutdown, at the Heavy Ion Accelerator Facility (HIAF, Australia). New chemical extraction and measurement techniques, including improved non-isotopic normalisation, enabled detection limits as low as 0.6 femtograms (fg) per sample for Antarctic snow, and enhancement of precision from 30% (GAMS) to 16% (HIAF). The rather high concentrations of about 280 fg (^99^Tc) per g (dry mass) measured in peat indicate Tc accumulation in this archive, which opens the possibility of studying its migration behaviour even under reducing conditions. Furthermore, it allowed deduction of an improved estimate of the global ^99^Tc inventory to (120–190) TBq. The first direct detection of ^99^Tc at 8 fg L^−1^ in river water highlights the need for further studies in urban areas to evaluate potential contributions from nuclear medicine. The achieved sensitivity allows monitoring of on-going releases, improving the risk assessment of releases from nuclear waste repositories and tracer applications in environmental sciences.

## Abstract 2

Ultra-sensitive analysis of technetium-99 established a global fallout baseline in environmental reservoirs unaffected by direct releases, such as ocean and river water, and revealed accumulation of this fission product in peatlands.

## Graphical abstract

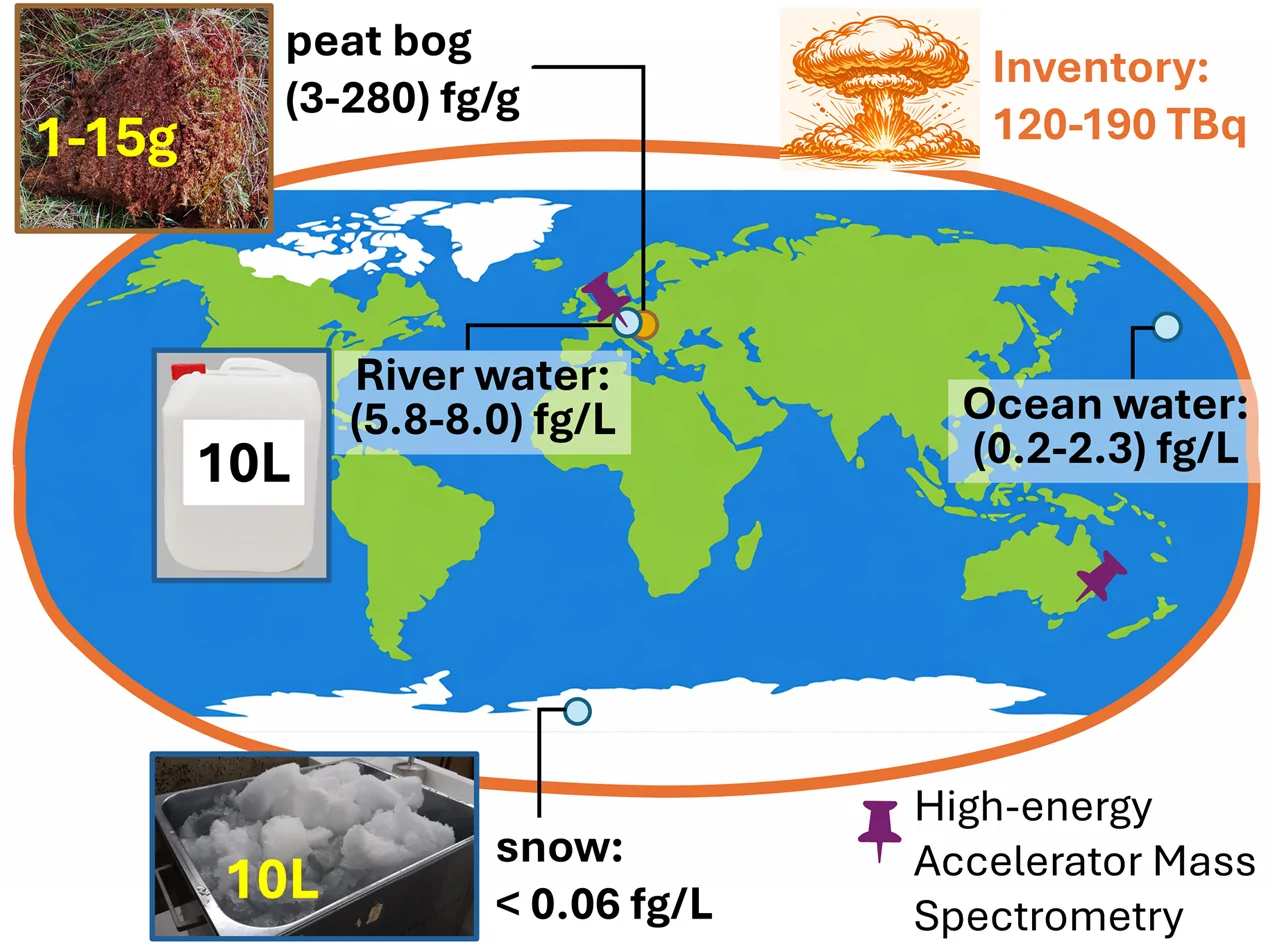

**Environmental significance**

Data on the environmental distribution of Technetium-99 (^99^Tc), a key radionuclide for nuclear waste management and a potential emerging contaminant from nuclear medicine, remains limited. By optimizing accelerator mass spectrometry procedures, this research established the global fallout baseline in environmental reservoirs that are not affected by direct releases and identified a ^99^Tc accumulation in peatlands. The reduced sample size required for this analytical approach, supports high-resolution monitoring of environmental ^99^Tc concentrations, essential for evaluating long-term environmental risks from nuclear medicine emissions or post-Fukushima contamination. Such studies will target surpluses above the established global fallout baseline and elucidate the migration behaviour of ^99^Tc.

## 1 Introduction

Considerable amounts of the long-lived fission product technetium-99 (^99^Tc, *t*_1/2_ = (2.111 ± 0.012) × 10^5^ years)^1^ have been discharged in the form of the highly soluble and thus mobile pertechnetate ion (TcO_4_^−^) predominantly from the Sellafield Reprocessing Plant (RP) to the Irish Sea (1.7 PBq, equal to *ca.* 2.67 t)^2^ between 1995 and 2005. This signal has been proposed as an oceanographic tracer for the North Atlantic owing to the predicted conservative behaviour of TcO_4_^−^.^3,4^ To determine whether the conservative behaviour of ^99^Tc is maintained under reducing conditions and/or in the presence of high concentrations of organic matter and complexing agents^5^ requires a comprehensive data set on the ^99^Tc distribution in real environmental systems with different chemical conditions (pH, Eh). Understanding Tc migration is also important from the perspective of bioaccumulation in the food chain as it has already been shown that Tc strongly accumulates for example in certain types of seaweed.^6–8^ Furthermore, profound knowledge on the migration behaviour is important in the context of nuclear waste storage^9^ and to assess the impact of widespread use in nuclear medicine on the occurrence of ^99^Tc in the environment.^10^ Technetium-99 has been also released on a global scale by atmospheric nuclear weapons testing and hence, has entered various compartments of the environment, in this way allowing for the study of its migration behaviour *in situ* and in a variety of different geochemical conditions. A comparably low total ^99^Tc deposition of 140 TBq (220 kg) from global fallout was estimated based on its relative fission yield to ^137^Cs.^11^ Data from direct measurements of ^99^Tc concentrations in environmental reservoirs affected by nuclear weapons fallout is scarce^12–14^ due to analytical difficulties. Radiometric detection of global fallout ^99^Tc levels of less than 0.05 mBq per g (soil)^14^ or 2 mBq per m^3^ (water)^12,15^ requires large sample masses/volumes because of the low specific activity.^7,16–18^ Mass spectrometric techniques are challenged by isobaric background, in particular ^99^Ru, and the lack of a suitable isotopic spike for normalisation. The well-established inductively-coupled plasma mass spectrometry (ICP-MS), for which detection limits of 10^10^ atoms ^99^Tc per sample (1.5 mBq) have been reported, suffers from interferences from neighbouring Ru and Mo isotopes and molecules such as ^98^Mo^1^H.^19,20^ Thermal as well as resonance ionisation mass spectrometry showed higher selectivity at the expense of a more tedious sample preparation.^21,22^ They have therefore rarely been used for the analysis of environmental materials with considerable matrix, *i.e.* large amounts of non-volatile and non-decomposable compounds. Even though Accelerator Mass Spectrometry (AMS) has achieved promising detection limits of 10^6^–10^7^ atoms per sample,^23^ in the past it has so far only been applied to rather concentrated reference materials^24^ or environmental samples with a low matrix.^25^ The biggest challenge for AMS as a relative detection technique remains the absolute determination of concentrations. Recently, research focused on using ^93^Nb for normalisation. This approach and a detailed comparison of analytical challenges regarding the detection of ^99^Tc especially by mass spectrometric techniques can be found in refs. 25 and 26. The aim of the present work is to extend these proof-of-principle studies to the application of AMS for the analysis of ^99^Tc in small-volumes of environmental samples (around 10 L) from a variety of archives that were not exposed to direct emissions from the nuclear industry. Establishing baseline levels of ^99^Tc in different compartments of the environment caused by fallout from nuclear weapons testing is essential for tracking recent input, *e.g.* from nuclear medicine. The selected sample materials including ocean and river water, Antarctic snow as well as peat moss exhibit different physico-chemical conditions and variable macroscopic matrix components, which can allow studying the ^99^Tc inventory and behaviour in these environmental systems, but at the same time require the systematic development of new AMS measurement and sample preparation protocols. In fact, the ocean water from the Northeast Pacific Ocean represents oxic conditions, high salt load and alkaline pH, while the peat bog shows reducing, anoxic conditions with high organic content and acidic pH. Global fallout from atmospheric nuclear weapons testing was expected to be the main source of ^99^Tc for all samples. Direct releases from the Fukushima Daiichi Nuclear Power Plant (FDNPP) accident are considered negligible for the Tc concentration in the Pacific Ocean water samples. However, ^99^Tc is present in the waste water storage tanks at concentrations of 0.8 Bq L^−1^ (ref. 27) as it cannot be fully removed by the ALPS (Advanced Liquid Processing System).^28^ Monitoring of ocean water after the discharge of ALPS treated water is considered necessary^27,29^ but requires sensitive detection methods as developed in the present study. This work demonstrates that the analytical procedures developed for AMS measurements using the gas-filled magnet setup of the Technical University of Munich (TUM, Germany) at the Maier-Leibnitz-Laboratory (MLL) were successfully adapted and further optimized for the different AMS setup at the Heavy Ion Accelerator Facility (HIAF) at the Australian National University (ANU, Australia). The transfer of the project from the MLL to HIAF became necessary when the MLL was closed at the end of 2019.

## 2 Materials and methods

### 2.1 Isotope spikes and reference material

The developed chemical and measurement procedures were verified using samples spiked with defined quantities of certified ^99^Tc solution (Eckert & Ziegler Nuclitec E&Z, NH_4_TcO_4_ in H_2_O) and the International Atomic Energy Agency (IAEA) reference materials Irish Sea water (IAEA-381/443)^30,31^ and sediment (IAEA-385).^32^ However, only information values have been published for the ^99^Tc concentrations of these IAEA-materials.^25,30,31^ Acceptable amounts for AMS, *i.e.* about 5 × 10^10^ atoms per AMS sample, corresponded to only 20 mL of IAEA-381. An in-house ^95m^Tc (*t*_1/2_ = (61.84 ± 0.07) d)^1^ spike solution was used as chemical yield tracer for sample preparation (see SI 2.1).

### 2.2 Sample collection and pre-treatment of environmental samples

As the analytical procedures were still being developed during measurements at MLL, samples are assigned to two different batches in the following depending on whether they have been analysed at the AMS facility at the MLL, Germany (batch 1) or at the HIAF, Australia (batch 2).

**Fig. 1:**
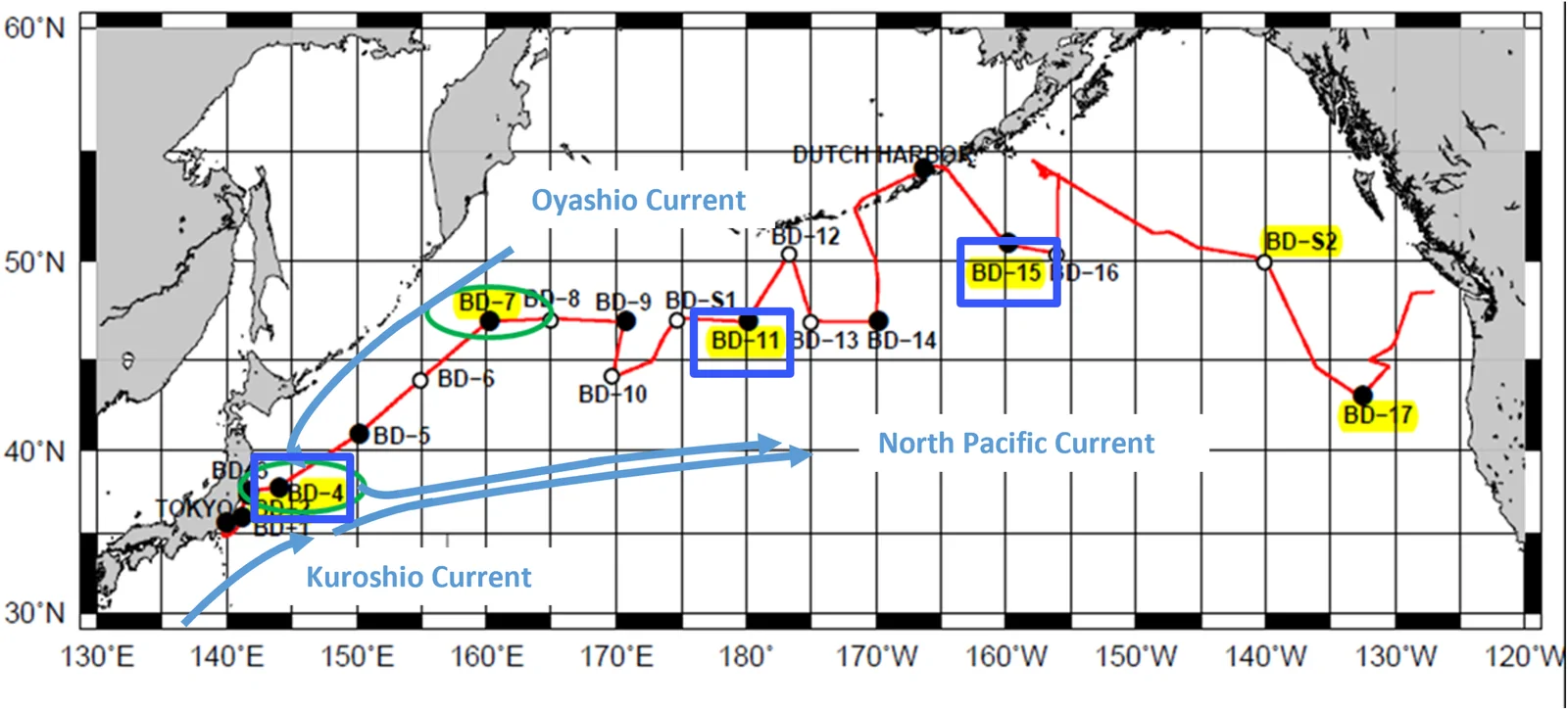
Sampling locations of cruise KH-12-4 in the North Pacific Ocean. The cruise track from Tokyo to Vancouver (August 23rd, 2012 to October 2nd, 2012) is presented as the red line. Samples analysed in this study are marked by green ovals (batch 1) and blue squares (batch 2). Blue arrows indicate the major flow direction of relevant ocean currents.

Water samples were collected in the North Pacific Ocean at sampling stations BD-4, BD-7, BD-11 and BD-15 (see Fig. 1) by the GEOTRACES cruise KH-12-4 (GP02) in 2012. Details on the sampling procedure along with the depth profiles of long-lived actinides at BD-4 have been published before.^33,34^ It is important to note that the samples have been acidified to pH 1 with HNO_3_ directly on board of the ship. Detailed information on individual samples from specific depths and their assignment to batch 1 or 2 can be found in SI 1.1. Sampling station BD-4 is located around 250 km off the Japanese coast where the Oyashio Current coming from the North and the Kuroshio Current from the South meet forming the eastward directed North Pacific Current leading to mixed water masses at BD-11 and BD-15 due to the subpolar gyre. In contrast, sampling station BD-7 is affected by the Oyashio Current only. With no additional known sources for ^99^Tc in the Kamchatka region, the Oyashio Current is supposed to transport mainly global fallout whereas in the Kuroshio Current contributions from close-in fallout from the Pacific Proving Grounds (PPG) can be expected. The corresponding ^240^Pu/^239^Pu^35–37^ and ^236^U/^238^U^38^ signatures of the high yield weapons tests at the PPG^39^ have been found in sediment and water as well as in corals off the Japanese coast.

Two ∼10 L (8.5 kg and 9.2 kg) water samples from the Isar River were collected in 2020 at separate locations downstream of Munich, *i.e.* near the town of Ismaning and the TUM research center Garching, respectively. The research reactor FRM II, a potential source for ^99^Tc, is located between the two sampling stations. For comparison, another 10 L (8.7 kg) water sample was collected in January 2023 from the Danube channel downstream of Vienna shortly before the confluence with the Danube River. All river water samples were collected from the water surface and downstream of the major wastewater treatment plants of both cities, filtered in the lab and acidified with HNO_3_ to pH = 1.

A total of 10 L (10 kg) of molten and filtered surface snow from Antarctica collected within a project on interstellar ^60^Fe at the German Kohnen Station (75°00′ S, 0°04′ E at 2892 m a.s.l.)^40^ was provided for this project. See ref. 40 for details on the location, sampling and sample pre-treatment.

Peat samples were collected in 2019 and 2020 in the mires Pürgschachen Moor (47°34′53″ N, 14°20′51″ E, *ca.* 630 m above sea level) and Rotmoos (47°41′0″ N, 15°9′18″ E, 690 m above sea level), both located in Styria, Austria. A description of the Pürgschachen Moor (PSM) sampling site, a continental raised bog, is available *e.g.* in ref. 41. The Rotmoos/Weichselboden (RM) is an undisturbed ombrotrophic peat bog with a clearly visible raised dome.^42^

Samples comprised three cut out peat blocks from PSM (batch 2) with dimensions (20 × 20 × 10) cm^3^ and two similar blocks from RM (batch 1) collected at different depths as well as two peat cores from 0 cm to 50 cm and 50 cm to 100 cm, respectively, from RM (batch 2). Slices of about 1 cm thickness were cut from the frozen peat blocks and of 3 to 5 cm from the peat cores. While 10 g and 15 g, respectively, was used for batch 1, only one gram of the dried and homogenized peat was processed further for each AMS sample of batch 2. Details on sampling and pre-treatment of the material can be found in SI 1.2.

### 2.3 ^99^Tc sample preparation for AMS

Supra-pure reagents from Carl Roth GmbH+ Co. KG (ROTIPURAN™ Supra) or from VWR Int. GmbH (NORMATOM™) were used for sample preparation. Higher purity grades like “ultra-pure” did not improve the Ru background.^26^ Corresponding process blanks were prepared from Milli-Q water (>18 MΩ) in parallel with the ocean water samples and from 5 mL HNO_3_ conc. for the peat samples as used for microwave digestion to monitor any background originating from the reagents and the preparation of the samples. A Ru-spike (diluted from ULTRA Scientific 1000 µg g^−1^) of 10^13^–10^14^ atoms per sample was added to both types of procedure blanks used in batch 2 (see Section 2.6).

#### 2.3.1 Water samples

**Fig. 2:**
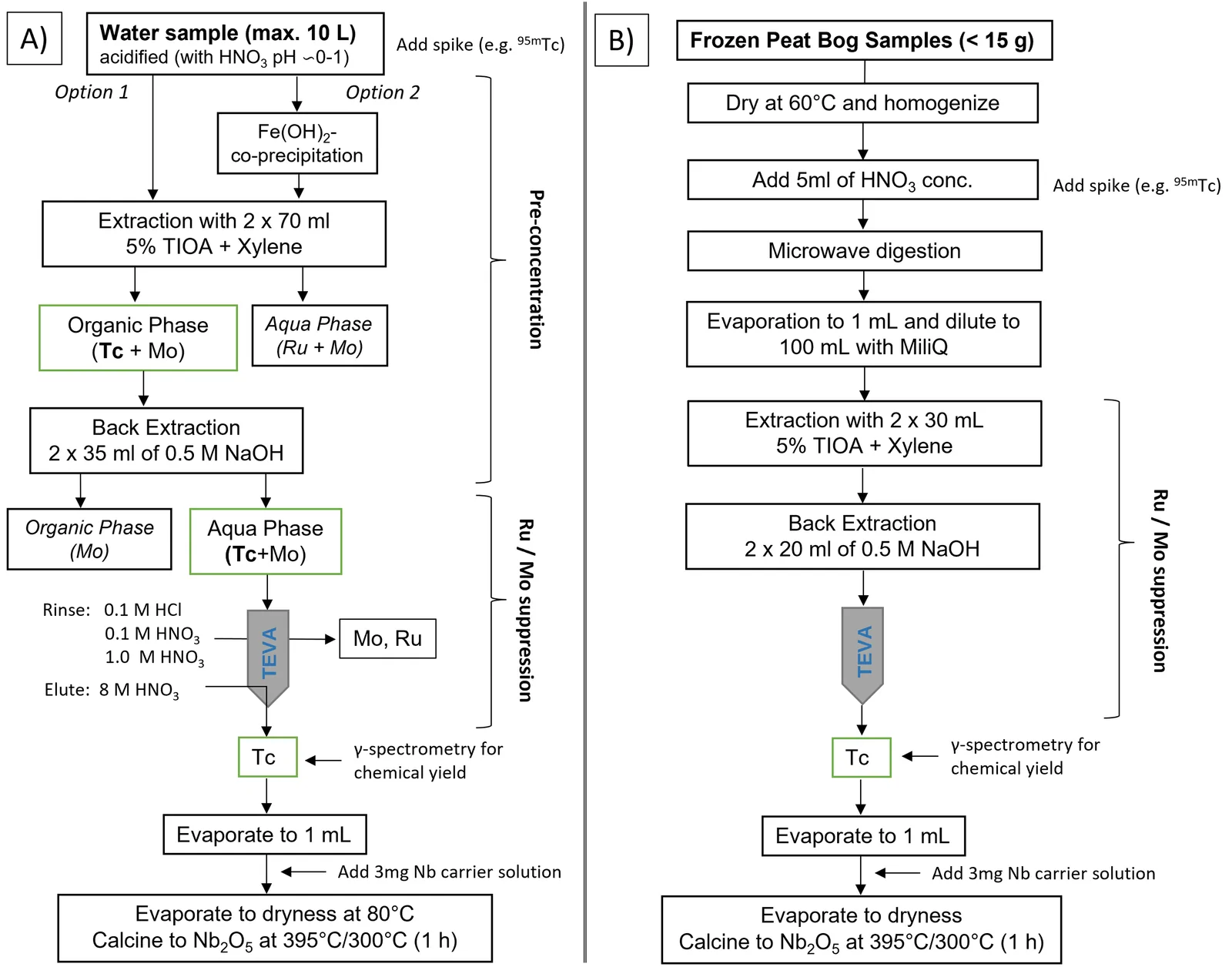
Chemical sample preparation for (A) ocean water samples with high salt load and (B) peat bog samples with high organic content. Ru/Mo suppression with TEVA for peat bog samples in (B) follows the same protocol as in (A). A full description of the chemical procedure is given in SI 2.

For the efficient extraction and purification of Tc from the large volume sea water samples, existing procedures (as summarized in ref. 2) based on Liquid–Liquid-Extraction (LLX) with 5% TIOA–xylene^43^ and extraction chromatography using TEVA® resin (*e.g.* ref. 24) were combined and adjusted to the requirements of AMS. The final procedure applied to the 10 L water samples is presented in Fig. 2A. A detailed description of the procedure is given in SI 2.2. The ^95m^Tc yield monitor was directly added to the initial water sample but was not available, yet, for the initial sample batch BD-4 0–400 m. For this sample batch, an average chemical yield of (89 ± 6)% (marked by * in SI Table 1), calculated from the subsequent ocean water samples, was applied. While the Tc extraction by LLX works efficiently (option 1 in Fig. 2A), the application to large volumes is very tedious. Therefore, a pre-concentration step using Fe(OH)_2_ co-precipitation was tested to reduce the volume (option 2), which, unfortunately, substantially decreased the overall Tc recovery to about 33–60% for the corresponding samples of batch 2 (compare Table 2). We attribute this to the acidification of the sample with the oxidizing acid HNO_3_. After the supernatant was discarded, the precipitate was dissolved again by adding a few mL of HNO_3_ and LLX was performed with the resulting 1–2 L sample volume in addition as preliminary test experiments showed an increased Ru suppression when LLX was used (see SI Fig. 1). The ^95m^Tc recovery determined with gamma spectrometry generally was ≥80% for 10 L ocean water samples when using option 1 (compare Table 2).

#### 2.3.2 Peat samples

Information on Ru concentrations in peatlands is limited with the exception of a thesis, which reported quite variable concentrations between 0.024 and 2.3 ppb in different peat layers.^44^ We therefore combined the pre-treatment method from ref. 25 using microwave digestion to oxidize the organic matter component of these samples with the Tc purification procedure developed for the sea water samples including the LLX step (see Fig. 2B) for enhanced Ru suppression (see SI 2.3). For batch 1, the solid residue after filtration was processed further, for batch 2 no separation of the pore water from the peat was performed. The ^95m^Tc spike was added to the sample in concentrated HNO_3_ before microwave digestion to achieve a homogenous mixture. Chemical yields obtained with this procedure were also above 80% (compare Table 3), except for one sample, PSM5-15/2 which was accidentally evaporated to dryness at 200 °C, resulting in a substantially lower recovery of only 42%. This observation confirms previous reports on ^99^Tc losses for evaporation to dryness.^2^

#### 2.3.3 Preparation of the AMS sample

The eluate from the TEVA column (see SI 2.2 and 2.4) was evaporated to 1 mL, first at 80 °C (batch 1) and later, at 200 °C (batch 2), after no ^99^Tc losses were observed in dedicated yield measurements for nitric acid solutions using the ^95m^Tc tracer (see SI 2.5 and SI Table 3). An in-house Nb-solution (*c* = 1 mg mL^−1^) was added and processed further as described in ref. 34 to obtain about 4 mg niobium oxide matrix for each AMS sample, which was finally pressed into Cu AMS sample holders. External standards for normalisation were in general prepared following the same steps after adding a defined amount of the Eckert & Ziegler (E&Z) ^99^Tc solution. To monitor the reproducibility of the entire procedure (preparation + measurement), four standards for batch 2 were prepared from 1 L of Milli-Q water and treated with the sample preparation protocol according to option 1 in Fig. 2A.

### 2.4 ^99^Tc analysis by AMS with a gas-filled magnet (GAMS, MLL)

The efficient ^99^Ru suppression of the unique GAMS setup using the 14 MV tandem accelerator of the MLL allowed the first ^99^Tc detection in low matrix environmental samples (with a maximum of *V* = 100 mL) at the level of 1 × 10^6^ atoms per mL.^25^ It was shown that the absolute ^99^Tc concentration in the initial environmental sample can be determined with a reproducibly of ∼30%,^23^ when a beam of stable ^93^Nb^11+^ extracted as ^93^NbO^−^ from a Nb_2_O_5_ matrix and an external standard with known ^99^Tc/^93^Nb ratio is used for normalisation. Details on the non-isotopic normalisation are described in ref. 25. A similar approach was chosen for the present study, also extracting ^99^Tc^16^O^−^ from the single-cathode Cs sputter ion source which then was stripped to charge state 12+ at a terminal voltage of 11.5 MV and hence accelerated to an energy of 148 MeV. We therefore conservatively assume that the minimal achievable accuracy and hence, uncertainty of this method is determined by the reproducibility of 30% mentioned above,^23^ since no additional quality assurance measures could be carried out within the remaining operation hours of the MLL.

The general GAMS setup with its gas-filled magnet and the 5-fold segmented ionisation chamber as detection system has been described in detail before along with the general settings used for the analysis of ^99^Tc^12+^.^23,25,45,46^ In contrast to previous work, the diverse set of environmental samples of this study showed a strongly varying background from Mo isotopes depending on the composition of the matrix, which made it necessary to use a time of flight (ToF) measurement in addition (compare SI 3.1 for further details). The ToF allows the highly abundant Mo isotopes to be clearly separated, while the stable isobar ^99^Ru is separated from ^99^Tc based on the different deflection in the magnet and their differential energy loss in the 5-anode ionisation chamber of the GAMS (see SI 3.1 for further details on the data evaluation). Narrow software cuts required for the identification of the ^99^Tc^12+^ counts, led to an acceptance of only about 1% of the ^99^Tc^12+^ counts with respect to the last beam current measurement used for normalisation to ^93^Nb^11+^ directly in front of the ToF path. The calculation of the ^99^Tc content of the sample starting from the counts in the region of interest (ROI) for ^99^Tc, including normalisation to the Nb^11+^ current and correction of the efficiency by normalisation to the external ^99^Tc standards, as well as a tuning specific Ru correction determined with Ru standards, followed closely the procedure published in ref. 25. Its application to the present measurements can be found in SI 3.1 and 3.2.

### 2.5 ^99^Tc analysis by AMS with an 8-anode ionisation chamber (HIAF)

First, promising attempts to analyse environmental ^99^Tc using the AMS setup of the HIAF at the Australian National University with the 14 UD pelletron tandem accelerator and an eight-anode ionisation chamber have been reported by Wacker *et al.*^24^ In the present study, a terminal voltage of ∼13.6 MV was used and the 13+ charge state with a predicted charge state yield of 11.3% was selected for Tc, resulting in a total energy of 189 MeV. The suppression of neighbouring masses at HIAF is very high owing to the exceptional energy resolution of (0.6–0.7)% at this particle energy (compare ref. 47) so that no additional separation *e.g.* by time-of-flight measurements like for the GAMS setup at MLL was required to remove corresponding background. For tuning of the AMS-system and normalisation of the measurements, the procedure developed at the MLL, using ^93^Nb, extracted as ^93^NbO^−^, was adopted using charge state 12+ on the high energy side. However, environmental samples which had been analysed at the MLL could not be re-measured to check reproducibility or to improve counting statistics as the sample holder design for the respective ion sources is not compatible.

The total energy (*E*_tot_) as well as the energy loss signals from each of the eight anodes of the detector (Δ*E*_1_ − Δ*E*_8_) were recorded for each event in the detector which allow for the separation and identification of ^99^Tc from its isobar ^99^Ru. The detector gas pressure was adjusted so that only ∼5% of the ^99^Ru ions reached the last anode of the detector. In contrast, ∼65% of the ^99^Tc ions reached the last anode under these conditions. Software cuts on the ^99^Tc peaks in the Δ*E*_2_, Δ*E*_6_ − Δ*E*_8_, and *E*_tot_ spectra were defined using standard and blank samples. A 2-dimensional spectrum of *E*_1–5_ (sum of signals Δ*E*_1_ − Δ*E*_5_) *vs. E*_6–8_ of events allows the definition of a region of interest to accept as many of the ^99^Tc events as possible (typically around 5%) while rejecting almost all ^99^Ru events (see SI Fig. 3).

Procedures for data evaluation were adapted from the GAMS measurements according to SI 3.2, but the Ru correction factor, determined from the ^99^Ru integration area defined in the ungated *E*_1–5_*vs. E*_6–8_ spectrum, was determined multiple times for each machine tuning to improve the accuracy of the correction. Furthermore, the normalisation factor was calculated as the average from three individual standard samples measured with the same machine tuning to quantify the reproducibility (compare SI 4.2).

### 2.6 Establishing the sensitivity for ^99^Tc

For most of the existing mass-spectrometric methods, including AMS, the achievable blank level for ultra-trace detection of ^99^Tc in environmental samples critically depends on the Ru-content of the sample. As process blanks are, however, usually prepared from ultra-pure solutions, the sensitivity and hence, the limit of detection (LOD, 3 sigma over blank level) deduced from them with a given setup are not representative for environmental samples. For the measurements at HIAF, Ru count rates differed by factor 15 (see Table 2), for example. While the Ru correction factor assumes a constant fraction of the Ru-counts to be scattered into the ROI, this proportionality is less well fulfilled for large differences in Ru rate. Consequently, pure blanks tend to be overcorrected and a formal subtraction of the overcorrected blank thus potentially would overestimate the true ^99^Tc concentration in samples with high Ru content. While an average Ru correction factor was sufficient for the first measurements at the MLL where Ru is strongly suppressed by the GAMS setup, we tested a different approach to obtain a more representative blank level and hence, achieve equivalent or better accuracy with the ionisation chamber at HIAF. For this purpose, 9 × 10^12^ and 9 × 10^13^ atoms of ^99^Ru were added to a process blank of batch 2 respectively corresponding to the 10 L ocean water samples, in which Ru is expected to be present at a concentration of 8 × 10^11^ atoms per litre.^48^ This approach was not possible for the peat bog samples, as Ru concentrations are not sufficiently well known for this reservoir.^49^ All blanks were then Ru corrected using an average Ru-correction factor derived from the respective tuning and further evaluated analogously to the environmental samples. The ^99^Tc/^99^Ru separation at HIAF is based on the small relative difference in energy loss in the gas of the ionisation chamber in contrast to the active Ru suppression which was provided by the GAMS setup. Consequently, the Ru correction in the case of HIAF measurements has been found to be significantly higher than for the measurements with GAMS (35% *vs.* 5% for the ocean water sample with the highest ^99^Tc concentration of each depth profile).

Apart from the background caused by Ru, we possibly introduce ^99^Tc into the sample by the addition of the ^95^Tc-spike in which ^99^Tc may be co-produced during the irradiation process. A conservative approach to establish a sensitivity limit taking the different sources of background into account is to analyse an environmental sample of similar material in which negligible amounts of ^99^Tc are expected to be present. The surface snow sample from Antarctica, for which a contribution from global fallout radionuclides was estimated to be negligible^40^ served this purpose in the present work.

## 3 Results and discussion

If not stated otherwise all results are presented with an uncertainty of one sigma. Sensitivity limits are given as the upper limit corresponding to one sigma uncertainty which is consistent with a zero measurement. Results are given in number of ^99^Tc atoms in the sample (^99^Tc at) or in number of ^99^Tc atoms per (kilo)gram (at per (k)g).

### 3.1 Reference materials and standards

The results obtained for the reference material IAEA-381 and the spiked samples agree reasonably well with the reference values as shown in Table 1 given the considerable spread of the published values and that no ^95m^Tc chemical yield monitor had been available for these first tests. Tc losses during sample preparation were assumed to be negligible for the small volumes used here. There is no significant difference between the results for the reference samples prepared by different chemical purification methods. The small volume of the reference material which can be used without risking cross-contamination obviously introduces little potentially interfering Ru and competing matrix elements which would require the additional Ru suppression by LLX. The measurement uncertainties of the spiked samples match or even exceed the reproducibility of 30% observed previously,^23^ while the uncertainties of the reference material samples are a factor of 2 lower. The lower precision of the results for the two ^99^Tc spiked MQ-water samples was caused by a strong increase of the ^93^Nb current indicating the importance of stabilizing the source output before the start of the measurement when using non-isotopic normalisation. The assumption of a minimum uncertainty of 30% for measurements of unknown samples at the MLL however seems justified.

**Table 1:** Results for reference materials (IAEA-381 and IAEA-385) and standards measured in comparison to nominal values and the corresponding process blanks

Type	Mass (g)	Preparation method	AMS facility	Number of ^99^Tc atoms (10^8^ at)	^99^Tc concentration (10^8^ at per g)	Nominal and literature values (10^8^ at per g)
IAEA-381 Irish Sea water	20.0 ± 0.5	LLX + TEVA (scheme A, option 1)	MLL	480 ± 50	24 ± 3	20.8 ± 1.1,^30^ 19.1 ± 3.6 (IAEA-443),^31^ 22 ± 6,^25^ 18.2 ± 2.6 ^50^
	20.0 ± 0.5	TEVA	MLL	530 ± 60	26 ± 3	
IAEA-381 Irish Sea water added to Milli-Q	2.5204 ± 0.0001 in 500 mL Milli-Q	LLX + TEVA (scheme A, option 1)	HIAF	90 ± 23	36 ± 9	
MQ-water spiked with ^99^Tc	20.0 ± 0.5	LLX + TEVA (scheme A, option 1)	MLL	3.3 ± 1.5	0.17 ± 0.08	0.25 ± 0.01
	20.0 ± 0.5	LLX + TEVA (scheme A, option 1)	MLL	6.4 ± 1.8	0.32 ± 0.09	0.27 ± 0.01
IAEA-385 Irish Sea sediment	0.1069 ± 0.0001	Microwave + LLX + TEVA (scheme B)	HIAF	27 ± 7	250 ± 60	67–350 ^32^
Process blank 1	20.0 ± 0.5	LLX + TEVA (scheme A, option 1)	MLL	<0.058		
Process blank 2	20.0 ± 0.5	TEVA	MLL	<0.032		

A reasonably good agreement with the published literature values within at least two sigma was also achieved for results obtained at HIAF for the two reference materials IAEA-381 and IAEA-385. However, the precision of the results in these initial measurements was rather low, *i.e.* up to 27%, which is also comparable to the results from the MLL, owing to a considerable scattering of the standards. During the measurement of the environmental samples, a reproducibility of 16% for the external standards was achieved when applying pre-sputtering of all samples for 10–15 minutes (see SI 4.3 for details) before actual start of data collection. Accepting these elevated uncertainties, the normalisation to ^93^Nb therefore represents a feasible approach for AMS to obtain data for ^99^Tc concentrations at highest sensitivity.

### 3.2 Sensitivity limit for ^99^Tc

Blank levels obtained from the process blanks representing the sensitivity limit of the measurement are listed together with the corresponding batch of samples in Tables 1–3. Generally, the high sensitivity of less than 5 × 10^6^ atoms per sample achieved by the GAMS setup reported in previous studies^23,25^ is confirmed even for chemically processed samples (see *e.g.*Table 1). Since the Ru suppression power of the GAMS setup is rather stable, the achievable blank level mainly depends on the Ru content in the AMS sample which is generally low for process blanks as pointed out in Section 2.7. Again, no significant difference is observed for the application of different preparation methods given the already very high purity of the reagents used with respect to Ru. More Ru might be introduced into the final AMS sample by the additional reagents used for the LLX leading to higher levels than if just using the TEVA separation. Both Ru-spiked “water-type” process blanks measured with the HIAF setup gave a comparable upper blank limit of <8 × 10^6^ and <4 × 10^6^ atoms per sample, respectively, despite the difference in Ru concentration of one order of magnitude indicating robustness of the average Ru correction in this concentration regime. The combined value from both blanks of <4 × 10^6^ atoms per sample is consistent with the upper limit of <3.7 × 10^6^ atoms of ^99^Tc per sample obtained for the Antarctic snow sample (see Table 2). We interpret this result as the general sensitivity which can presently be reached for real environmental samples with the AMS setup at HIAF. It translates into an excellent LOD of 1.1 × 10^7 99^Tc atoms or 1.1 µBq per sample.

**Table 2:** Measurement results for ^99^Tc in environmental waters, *i.e.* Pacific Ocean and river water, and snow, including Tc recovery during sample preparation and corresponding blank levels^a^

Sample name	Sample type	Tc recovery (%)	AMS facility	Collected counts in ^99^Tc ROI	^99^Ru count rate (s^−1^)	Ru corrected ^99^Tc counts	Number of ^99^Tc atoms (10^7^ at)	^99^Tc concentration (10^6^ at per kg)
BD-4-0 m	Pacific Ocean	89 ± 6*	MLL	29	0.6	26	6.2 ± 1.9	5.5 ± 1.7
BD-4-100 m	Pacific Ocean	89 ± 6*	MLL	14	0.2	11	0.9 ± 0.5	0.8 ± 0.4
BD-4-200 m	Pacific Ocean	89 ± 6*	MLL	95	0.8	89	7.7 ± 2.4	6.9 ± 2.1
BD-4-400 m	Pacific Ocean	89 ± 6*	MLL	149	0.9	142	11.8 ± 3.6	12.6 ± 3.9
Blank-4_0-400 m	Blank	89 ± 6*	MLL	3	0.4	0	<0.5	<0.5
BD-4-600 m	Pacific Ocean	77.9 ± 0.8	MLL	30	2.1	22	4.4 ± 1.9	3.8 ± 1.7
BD-7-0 m	Pacific Ocean	84.5 ± 1.0	MLL	9	1.5	1	<1.6	<2.0
BD-7-300 m	Pacific Ocean	94.8 ± 1.0	MLL	40	2.0	14	4.3 ± 2.2	4.2 ± 2.2
BD-7-600 m	Pacific Ocean	95.5 ± 1.0	MLL	13	1.2	9	3.0 ± 1.5	2.8 ± 1.4
BD-7-1000 m	Pacific Ocean	93.4 ± 1.0	MLL	20	1.6	11	<1.4	<1.2
BD-7-2000 m	Pacific Ocean	89.6 ± 1.2	MLL	5	4.6	0	<4.4	<2.0
BD-7-3000 m	Pacific Ocean	94.2 ± 0.8	MLL	9	1.2	1	<1.4	<1.6
BD-7-5000 m	Pacific Ocean	90.6 ± 1.3	MLL	15	2.1	1	<1.7	<1.6
Blank-07	Blank	92.3 ± 1.0	MLL	8	3.1	0	<0.7	<0.7
BD-11-0 m	Pacific Ocean	33.6 ± 1.4^(1)^	HIAF	94	18	64	9.7 ± 1.7	8.9 ± 1.6
BD-4-20 m	Pacific Ocean	37.1 ± 1.9^(1)^	HIAF	132	20	94	15.0 ± 2.5	14.2 ± 2.4
		45.2 ± 2.3^(1)^	HIAF		36			
BD-11-200 m	Pacific Ocean	60.7 ± 3.1^(1)^	HIAF	69	16	53	11.0 ± 2.0	8.7 ± 1.7
BD-15-600 m	Pacific Ocean	91.1 ± 4.1	HIAF	52	23	27	2.5 ± 1.1	2.5 ± 1.0
		79.2 ± 2.9	HIAF		11		2.5 ± 1.1	
BD-11-2000 m	Pacific Ocean	37.8 ± 1.6^(1)^	HIAF	17	11	2	<1.4	<1.2
Danube channel	River water	88.9 ± 4.1	HIAF	446	21	434	42.7 ± 4.4	49 ± 5
Isar-1	River water	83.0 ± 2.9	HIAF	503	21	488	32.2 ± 3.3	35.1 ± 3.5
Isar-2	River water	33.5 ± 1.3^(1)^	HIAF	188	52	129	39 ± 7	46 ± 8
Antarctic snow	Snow	91.8 ± 3.9	HIAF	19	32	0	<0.38	<0.37
Blank (9 × 10^13^ Ru)	Blank	83.2 ± 3.3	HIAF	10	42	0	<0.8	
Blank (9 × 10^12^ Ru)	Blank	93.7 ± 3.4	HIAF	14	19	0	<0.4	

a (*) average Tc recovery before the ^95m^Tc spike was available (see main text for further details). Counts collected in the region of interest for ^99^Tc (ROI) before and after Ru correction are given for comparison. Results from individual cathodes have been combined to obtain the number of ^99^Tc atoms in the initial environmental sample. ^(1)^ Indicates an additional Fe(OH)_2_ co-precipitation used for pre-concentration.

**Table 3:** Measurement results for ^99^Tc in peat bog samples from Pürgschachener Moor (PSM) and Rotmoos (RM) in comparison to the process blank including Tc recovery during sample preparation^a^

Sample name	Sample type	Tc recovery (%)	AMS facility	Counts in ^99^Tc ROI	^99^Ru count rate (s^−1^)	Ru corrected ^99^Tc counts	Number of ^99^Tc atoms (×10^8^ atoms)	^99^Tc concentration (×10^8^ atoms per g_dry_)
RM75_10 g	Rotmoos	82.4 ± 0.9	MLL	50	0.5	47	0.74 ± 0.22	0.09 ± 0.03
RM75_15 g	Rotmoos	89.4 ± 1.2	MLL	86	0.4	84	2.330 ± 0.070	0.170 ± 0.005
BLK-RM	Blank	92.0 ± 1.1	MLL	4	0.6	1	<0.05	—
PSM5-15	Pürgschachen	90.4 ± 3.5	HIAF	1499	10	1486	30.3 ± 2.3	15.2 ± 1.1
		41.9 ± 1.5	HIAF		33			
PSM15-25	Pürgschachen	93.2 ± 2.5	HIAF	1252	29	1235	18.2 ± 1.6	9.1 ± 0.8
		84.3 ± 3.4	HIAF		14			
PSM25-35	Pürgschachen	88.4 ± 3.6	HIAF	163	14	152	0.79 ± 0.17	0.79 ± 0.17
RM20-27	Pürgschachen	90.0 ± 3.4	HIAF	1658	13	1652	16.8 ± 1.6	17.1 ± 1.6
RM43-52	Pürgschachen	89.7 ± 1.7	HIAF	68	14	58	0.32 ± 0.08	0.32 ± 0.08
RM70-77	Pürgschachen	96.3 ± 4.4	HIAF	19	9	13	0.12 ± 0.05	0.12 ± 0.05
RM89-97	Pürgschachen	99.4 ± 3.5	HIAF	82	18	70	0.37 ± 0.08	0.38 ± 0.08
Blank (no Ru)	Blank	84.3 ± 2.9	HIAF	3	3.5	3	0.34 ± 0.24	
Blank (water-type, 1 × 10^14^ Ru)	Blank	83.2 ± 3.3	HIAF	32	71	14	0.2 ± 0.1	

a Results from individual AMS samples have been combined to obtain the number of ^99^Tc atoms in the initial environmental sample.

Both process blanks (Ru-spiked “water-type” blank and unspiked “peat-type” blank), which were analysed in the measurement campaign dedicated to the peat bog samples, gave similar blank values of (20 ± 10) × 10^6^ and (34 ± 24) × 10^6 99^Tc atoms per sample, respectively. Consequently, the blank level was about a factor 10 higher compared to the experiment on the water samples but again hardly dependent on the Ru concentration. This observation suggests cross-contamination of the unspiked blank with ^99^Tc, presumably during sample preparation, as the Ru count rate was almost 20 times lower than that of the spiked blank and the Ru correction factor was correspondingly small. No additional investigation of the reason for the higher blank level or optimization of the setup was required, as a pronounced ^99^Tc signal above the processing blank level was found in most peat bog samples.

### 3.3 The ^99^Tc signal in the Pacific Ocean

**Fig. 3:**
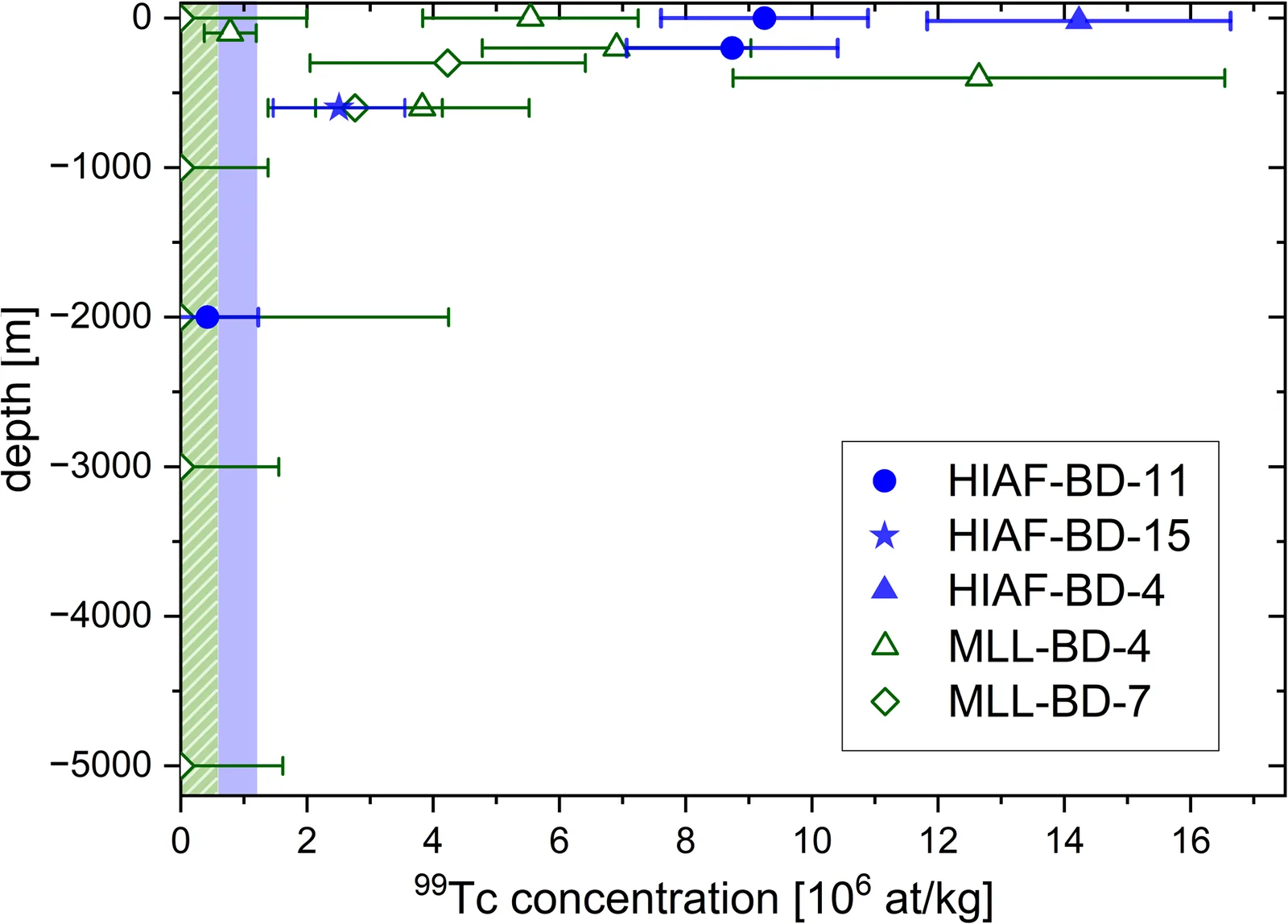
Depth profile of the measured ^99^Tc concentrations (not blank corrected) for Pacific Ocean water samples obtained at HIAF (blue, filled symbols) and MLL (green, open symbols). The blue and the (green) shaded vertical region mark the corresponding averaged 1 sigma upper limit of the blank level for measurements at HIAF and MLL, respectively.

Measurement results for the Pacific Ocean water samples including number of ^99^Tc atoms and the resulting ^99^Tc concentrations are presented in Table 2 along with the corresponding process blanks. In addition, the number of counts collected in the ^99^Tc ROI before and after Ru correction (calculation according to SI 3.2) are listed. Results from individual cathodes for a sample (compare SI 4.4 and SI Table 6) were combined by adding up the events or by averaging the concentrations. For the data from MLL, Ru corrections are generally small, not only for process blanks but also for the seawater samples and do not change the overall trend of higher concentrations towards the water surface and at station BD-4 compared to BD-7. Also for the data set from HIAF, the application of an average Ru correction factor, which is substantially higher than for the GAMS data as pointed out before, does not change the overall concentration distribution. The higher sensitivity limit obtained for the lowest water samples compared to the blank level was mainly a result of a lower source output compared to the blanks and the limited available measurement time. For the reasons detailed above, no formal blank correction was made in this initial approach, but signals from the individual environmental samples are discussed relative to each other. Consequently, the lowest sample from the Pacific Ocean depth profile, *i.e.* BD-11-2000 m for the HIAF data and BD-11-3000 m for the MLL data, represent a conservative estimate for the sensitivity reached in this measurement, and are considered as the pre-nuclear ^99^Tc baseline. The value of these samples are consistent with the blank level obtained with the Ru spiked process blank, indicating that the Ru background in 10 L ocean water samples is well represented by 10^13^–10^14^ Ru atoms. The corresponding depth profiles and blank levels are plotted in Fig. 3 comparing the results obtained by HIAF and MLL. Combining data from sampling station BD-11 with BD-4 and BD-15 obtained by HIAF represents an exemplary depth profile for the North Pacific Ocean. A maximum is observed at shallow depths with a concentration of (14.2 ± 2.4) × 10^6 99^Tc at per kg at a depth of 20 m and decreases for samples collected at greater depth. At the ocean surface and at 200 m depth similar intermediate concentrations of (9.3 ± 1.6) × 10^6 99^Tc at per kg and (8.7 ± 1.7) × 10^6 99^Tc at per kg, respectively, were measured. At 600 m depth, the concentration has already dropped to a value which agrees with the baseline value of BD-11-2000 m within two sigma. For station BD-7, only samples from depth of 300 m and 600 m show a signal but these also agree with a zero value within less than 2 sigma and are therefore considered as not significant. However, a significant signal could be detected for station BD-4 at a similar depth as for the two slightly elevated values of BD-7. In fact, higher concentrations at BD-4 compared to BD-7 are to be expected due to the transport of tropospheric close-in fallout from the Pacific Proving Grounds by the Kuroshio Current which reaches BD-4 but not BD-7.

Despite the substantial differences in instrumentation, both data sets show a good agreement over the range of measured concentrations, with a maximum of ∼1 × 10^7^ atoms ^99^Tc per kg close to the surface. This distribution with the maximum located within the first 100 m depth is similar to that of other conservatively behaving radionuclides originating from surface deposition such as ^137^Cs^51^ and also ^236^U,^52,53^ for which a depth profile from the same sampling location BD-4 has already been published.^34^ The combined data gives a consistent picture with a clear drop-off towards 1000 m depth, which is not compatible with the subsurface maxima at about 1000 m depth observed for particle-reactive elements like Pu in the Pacific Ocean.^33,34,54–56^ This observation supports a conservative behaviour of Tc, which agrees with the expected mobile behaviour of ^99^TcO_4_^−^ in which Tc should be present under oxidizing conditions. The maximum value at 400 m corresponding to a ^99^Tc activity of (1.32 ± 0.41) µBq kg^−1^ is also in very good agreement with the limited published data such as that from a sampling area north of BD-4, *i.e.* off the coast of Aomori Prefecture.^15^ The slightly higher concentrations reported in ref. 15 with a maximum of (2.96 ± 0.29) µBq L^−1^ between 30 and 100 metres depth, could be due to the greater proximity to the coast and thus, potential emission sources, such as the reprocessing plant in Tokai. Measured absolute concentration for the open North Pacific Ocean represented by BD-11-0 m of (9.3 ± 1.6) × 10^6 99^Tc at per kg, corresponding to (0.97 ± 0.17) mBq m^−3^, is well comparable to concentrations of (0.63–0.77) mBq m^−3^ measured in more than 2000 L surface water collected from the open ocean at sampling stations south of Japan.^12^ The higher precision of the HIAF data indicates a more distinct maximum closer to the surface, whereas the significant dip at 100 m (batch 1) found for sampling station BD-4 with the GAMS setup is not evident and therefore requires further investigation. A similar minimum was observed at this sampling station for ^236^U/^238^U but with the local minimum located at 20 m depth.^34^

In general, the rather small present data set and the substantial associated uncertainties do not allow a detailed discussion of the potential emission sources or migration/transport behaviour of ^99^Tc. However, it should be noted that previous studies used several hundred to thousands of litres^12,15,57^ for ^99^Tc detection in the same or nearby sampling areas. In the present study, using only 10 litres, *i.e.*, reducing the sample volume by a factor of 10–100, is a considerable improvement. This further enables more wide-spread studies, faster sample preparation and a higher sample through-put.

### 3.4 The ^99^Tc signal in fresh water

Concentrations in all three river water samples of around 43 × 10^6 99^Tc at per kg (see Table 2, lower part) were a factor three, *i.e.* significantly, higher than the Pacific Ocean water samples and the number of atoms in the AMS sample is a factor ∼100 above the Ru spiked blank. To our knowledge, existing data on ^99^Tc in river water so far only covers locations with potential local input sources and are at least 2 orders of magnitude higher: (0.2–4) × 10^11 99^Tc at per L were found in river water samples in the Ottawa river at the site of the Chalk River Laboratories,^58^ and (0.1–3) × 10^11 99^Tc at per L were measured in the Yenisei River affected by radioactive effluents of the Rosatom mining chemical combine.^59^ The elevated ^99^Tc concentrations in the present river water samples, far away from known local contamination sources but downstream of two big cities, could point to a possible signal from medical applications above the global fallout baseline. Clearly, a larger dataset from metropolitan areas and comparative samples collected upstream are needed to be able to exclude re-mobilized global fallout ^99^Tc as explanation for this finding. No significant difference in ^99^Tc concentration has been observed up- and downstream (Isar-1 and Isar-2, respectively) of the FRM II in the Isar river water, hence indicating no extra input of ^99^Tc to the environment from the operation of the FRMII reactor.

### 3.5 The ^99^Tc signal in a peat bog

**Fig. 4:**
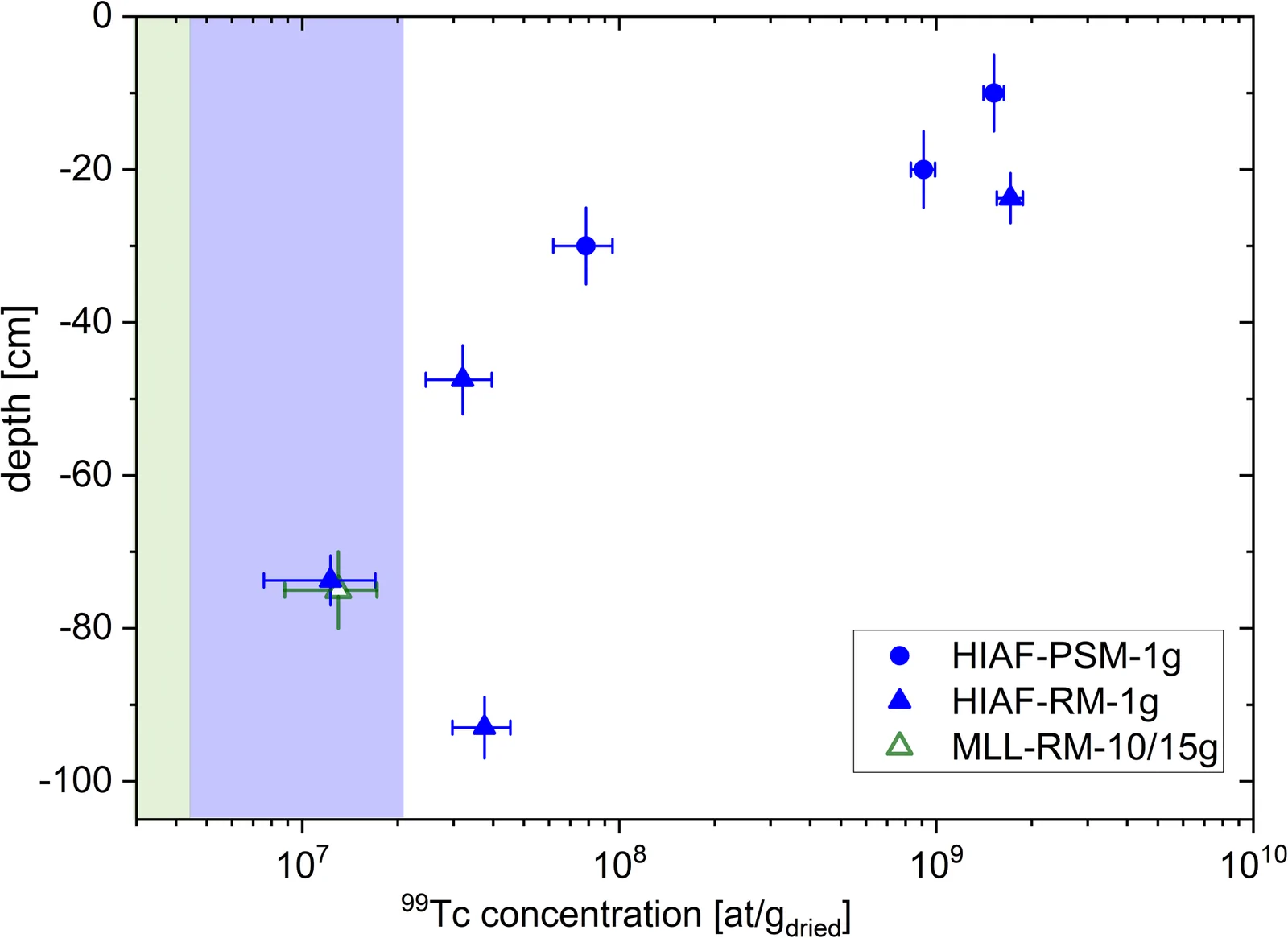
Comparison of the ^99^Tc concentrations per gram of dried peat (not blank corrected) obtained at HIAF (blue filled symbol) and MLL (green open symbols) from Pürgschachen Moor (PSM, circles) and Rotmoos (RM, triangle). Uncertainty in depth results from the thickness of the sample and sampling procedure. The average value of the 10 g and 15 g sample from batch 1 is plotted as the MLL result.

The measured number of ^99^Tc atoms and the corresponding concentrations per gram dry weight for the samples from RM and PSM along with the relevant process blank are listed in Table 3. Both peat samples from Rotmoos mire collected at the same depth (RM75_10 g and RM75_15 g) and analysed with the GAMS setup showed a clear signal above the corresponding process blank level in terms of number of atoms in the sample. The required Ru correction at a maximum 6% of the initial counts in the ^99^Tc ROI is small, indicating that the measurement suffered from only minor Ru background. The corresponding concentrations of (0.89 ± 0.27) × 10^7^ atoms per g_dry_ and (1.7 ± 0.5) × 10^7^ atoms per g_dry_, respectively, just agree within one sigma and thus result in a mean value of (1.3 ± 0.4) × 10^7^ atoms per g_dry_ (1.4 ± 0.5 µBq g_dry_^−1^) for the solid fraction of peat at a depth of 75 cm using the relative uncertainty as weights. This averaged value is plotted along with the concentrations obtained by the HIAF at their respective sampling depth in Fig. 4 and is in very good agreement with the HIAF result of (1.2 ± 0.5) × 10^7^ atoms per g_dry_ from the same depth. However, in contrast to the measurements at MLL, this sample defined the upper blank limit for the corresponding measurements at HIAF pointing to an overall lower sensitivity (see Section 3.2). It should be noted that the sample mass has been considerably decreased to only 1 g for the measurements at HIAF after the first successful detection of ^99^Tc in the solid peat with the GAMS setup. Even with these small samples, a first-time depth profile of anthropogenic ^99^Tc in a peat bog was obtained with the maximum concentration, most likely from global fallout, being almost two orders of magnitude higher than the background level at 47.5 cm to 93 cm depth. While the corresponding three samples at greater depth from the RM are compatible with the blank level of this measurement (compare Table 3), there is a pronounced signal between 5 cm and 27 cm. Highest ^99^Tc concentrations were found in the PSM at (10 ± 5) cm and in the RM at (23.8 ± 3.3) cm sampling depth (*i.e.* sample PSM5-15 and RM20-27) with (15.2 ± 1.1) × 10^8^ and (17.1 ± 1.6) × 10^8^ atoms per g_dry_, respectively. Consequently, maximum concentrations from both peat bogs are in good agreement with each other and would not be significantly changed by a blank correction using the lowest samples, *i.e.* below PSM25-35 at a depth of (30 ± 5) cm as a conservative estimate of the blank level for the peat. This result represents the first detection of ^99^Tc in a solid peat moss. The only other published value for peatlands of 1 × 10^6^ at per mL is for surface water of a peat bog lake in Southern Germany.^25^ Due to the different matrix of the material, however, direct comparison of the values is not possible. Combining all data from both mires (see Fig. 4) and with samples below a depth of (30 ± 5) cm defining the pre-nuclear baseline with 50–100 times lower concentrations, suggests that the maximum of the distribution is located in the upper layers between the surface and a depth of 30 cm. This corresponds well to the distribution of global fallout derived ^236^U in the ombrotrophic peat bog from the Blackforest, Germany, where the ^236^U/^238^U bomb peak from global fallout was found at a depth of 22 cm.^60^ The maximum ^99^Tc concentration agrees with the accumulated global fallout signals in uncontaminated paddy field soil of 6 × 10^7^ to 1 × 10^9^ at per g_dry_,^14^ which, considering the scarce global fallout data, is closest to peat in its sample material. In contrast, the maximum concentration found in the Austrian peat is 3–30 times lower than what has been reported for Polish peat samples affected by Chernobyl fallout.^61^ We therefore attribute the detected ^99^Tc signal to surface deposition of global fallout from atmospheric nuclear weapons testing but cannot exclude a contribution from the Chernobyl accident *per se*. Notably, the PSM was strongly affected by ^137^Cs deposition from the Chernobyl fallout compared to other regions in Austria.^62^ Depth profiles from less affected areas like the RM need to be analysed for comparison. A potential offset between the ^99^Tc distribution in the RM *versus* the PSM is indicated by the fact that the maximum concentration in RM was measured at a depth, *i.e.* 20 to 25 cm, where the concentration in PSM has already declined to (9.1 ± 0.8) × 10^8^ atoms per g_dry_. Alternatively, even higher maximum concentrations are expected to be reached in RM than in the PSM towards the surface, if the ^99^Tc concentration in the RM does follow the same distribution as in PSM. However, a detailed investigation of such a shift would require multiple peat cores from both mires to be collected following an established sampling protocol as described in ref. 63.

### 3.6 Estimation of global fallout ^99^Tc inventory

The depth distributions obtained with sample batch 2, *i.e.* Pacific Ocean water and peat bog, allows derivation of two independent estimates for the global fallout ^99^Tc inventory by integrating the profiles (see SI 5 for the details on the calculation). For the integration, we interpolated linearly between the data points, and the obtained areal concentrations were scaled with measured ^90^Sr deposition data, taking into account that nuclear weapons fallout was not distributed homogenously. In that way, the global inventory is calculated to (1.2 ± 0.5) × 10^27 99^Tc atoms, *i.e.* (120 ± 50) TBq from the ocean water column. As no measurement data is available for the surface of the peat bog, we distinguish between two limiting scenarios: (1) the ^99^Tc concentration decreases to background levels at the surface or (2) the surface concentration stays constant at the value of the closest data point (PSM5-15 cm). Following the same approach as for the water samples, we obtain for scenario (1) an estimate for the global inventory of (1.5 ± 0.5) × 10^27^ atoms ^99^Tc ((150 ± 50) TBq) and for scenario (2) (1.9 ± 0.6) × 10^27^ atoms ^99^Tc (or (190 ± 60) TBq). The estimated inventories are in the same order of magnitude for both scenarios and also for the two different depth profiles obtained from the peat bog and the Pacific Ocean. These values agree within their uncertainties with the estimate of 140 TBq based on ^90^Sr deposition data and the ^99^Tc/^137^Cs ratio for global fallout.^11^ Our estimate is in good agreement with the value that can be calculated from measurements of non-contaminated soil in Japan, where an area concentration of (0.47 ± 0.05) Bq m^−2^ (ref. 64) would lead to a global inventory of (115 ± 12) TBq. From integration over the different depth profiles reported in ref. 15, we derive an higher averaged estimate for the inventory of (240 ± 30) TBq. Our resulting estimates lie well within the range of these previously published values, confirming the detection of global fallout ^99^Tc.

## 4 Conclusions

The expertise and methodology developed at the MLL reaching the highest ^99^Tc sensitivities of about 5 × 10^6^ atoms per sample were successfully transferred to the HIAF, where procedures have been successfully adapted to the different detection setup and further improved. As demonstrated by the upper limit reached for the Antarctic snow sample, the HIAF approach is fully competitive with the GAMS in terms of sensitivity and provides even better separation of isotopic background, *e.g.* from the ubiquitous Mo isotopes. Application to an exemplary depth profile from the Pacific Ocean shows the feasibility of ^99^Tc detection in 10 L samples paving the way to comprehensive studies of the ^99^Tc distribution in the Pacific Ocean and to monitor possible input from the discharges of the FDNPP storage tanks to the ocean or from medical applications to the (urban) environment. The strong accumulation of ^99^Tc in the upper peat layers found in this study suggests the peat bog as a well-suited archive for surface deposited ^99^Tc similar to other contaminants. The lower sensitivity limit reached in the measurement at the HIAF, does not allow verification of whether the signal detected by the MLL at 75 cm depth indicates migration of global fallout ^99^Tc. However, with the successful detection of ^99^Tc in 1 g of sample material, the setup at the HIAF brings within reach a detailed investigation of a depth-resolved peat core. This allows more accurately determination of the contamination source distribution and provides an improved estimate of the environmental ^99^Tc inventory. Furthermore, it could also allow the study of the ^99^Tc migration behaviour in the specific geochemical conditions of a peat bog. When using peat cores with a similar diameter to those of the present study (6 cm), a depth resolution of ∼1 cm in the peak region is achievable. Assuming that the maximum ^99^Tc concentration at 25 cm depth corresponds to 1960 CE, this would result in a time resolution of about 3 years.

For quality control, however, reference materials with certified concentration values for ^99^Tc that are more than two orders of magnitude lower in concentration are required to be representative of the environmental samples targeted for analysis by AMS, and to avoid cross-contamination in the ion source or during sample preparation. Measuring unknown samples relative to such materials within each measurement campaign, as is routine for AMS nuclides like ^14^C, would substantially increase accuracy and precision of the measurements. Allowing equilibrium conditions to be established in the ion source before the measurement is started has already significantly increased the quality of the data. Further improvement can be expected from blanks spiked with variable amounts of Ru to reflect the Ru concentrations in the environmental samples which will then allow a formal blank correction. With the isobar separation being based on differences in the specific energy loss, the developed measurement procedure cannot be directly transferred to more compact AMS systems with terminal voltages below 10 MV which are more readily accessible. Following the closure of the MLL with its unique GAMS setup, this leaves the AMS setup at HIAF as the only option for highly sensitive ^99^Tc measurements in samples of practicable size. Ion-Laser Interaction Mass Spectrometry (ILIAMS) offers a possible solution here, as promising Ru suppression factors have been reported when extracting Ru as pentafluoride from the ion source. The achievable reproducibility using the normalisation to Nb for the extraction of fluoride compounds is the subject of intensive on-going research.

## Author contributions

KH: conceptualization, funding acquisition, methodology, investigation and supervision, writing – original draft. SA: investigation, formal analysis, visualization, writing – original draft. TF: methodology, investigation, writing – review & editing. LKF: methodology, investigation, writing – review & editing. MBF: methodology, writing – review & editing. FG: investigation. DK: methodology, investigation, formal analysis, writing – original draft. GK: conceptualization, methodology, investigation, writing – review & editing. MM: methodology, investigation and supervision, writing – review & editing. SP: methodology, investigation, formal analysis HIAF, writing – original draft. JP: investigation, formal analysis GAMS, data curation. FQ: methodology, sample preparation, investigation, writing – review & editing. GR: methodology, investigation, supervision. ST: methodology, investigation, resources, writing – review & editing. JW: methodology, supervision. AW: investigation. MY: resources.

## Conflicts of interest

There are no conflicts to declare.

## Supplementary Material

VA-005-D6VA00067C-s001

## Data Availability

The data sets for this article, including raw and evaluated data, can be found on: https://lehre4.rad.univie.ac.at/share/WWW_Exchange/public/Hain2026_99Tc_Low-Volume-Samples-Global-Environment-AMS/. Supplementary information (SI): sampling and sample preparation details as well as a description of the data evaluation process at the MLL and HIAF including background discrimination and raw data. Furthermore, results from quality control samples are discussed with respect to reproducibility and details on the calculation of the global ^99^Tc inventory are given. See DOI: https://doi.org/10.1039/d6va00067c.^99^Tc
