# Supplementary material for: Technetium-99 determination in low-volume samples from the global environment with accelerator mass spectrometry
Source: Env Sci Adv. 2026 Jun 5. Online ahead of print. doi: 10.1039/d6va00067c (PMC13267875; doi:10.1039/d6va00067c)
Supplement: VA-OLF-D6VA00067C-s001 [file VA-OLF-D6VA00067C-s001.pdf]

Supplementary Information (SI) for Environmental Science: Advances.
This journal is © The Royal Society of Chemistry 2026

## Supporting Information:

# Technetium-99 Determination in Low-Volume Samples from the Global Environment with Accelerator Mass Spectrometry

Karin Hain*, Stephanie Adler, Thomas Faestermann, L. Keith Fifield, Michaela B. Froehlich, Fadime Gülce, Dominik Koll, Gunther Korschinek, Martin Martschini, Francesca Quinto, Stefan Pavetich, Johanna Pitters, Georg Rugel, Stephen Tims, Jan Welch, Andreas Wiederin, Masatoshi Yamada

* Corresponding author

## 1 Sampling details

### 1.1 Water Samples

All Pacific Ocean water samples have been acidified to pH 1 with $HNO_3$ directly on board of the ship to prevent sorption of the actinides and $^{99}$Tc onto the walls of the sampling containers. Geographic coordinates, salinity, weight as well as Tc recovery for all water samples are given in SI Table 1. From the water column at sampling station BD-4 (see Fig. 1), which reaches down to 7000 m, only samples down to a depth of 600 m were available for this study. Selected samples from BD-4 were completely used up for a previous study on actinide concentrations [1]. The volume of the remaining samples was divided approximately equally between actinide and $^{99}$Tc analysis with 10 L each (see SI Table 1). For BD-7, samples down to the maximum depth of 5000 m were available for the present study and were grouped in batch 1 together with most samples from BD-4. The remaining BD-4 sample from 20 m depth was analysed alongside individual samples from BD-11, i.e. 0, 200 m and 2000 m, and BD-15 from 600 m depth in batch 2, representing an average depth distribution of $^{99}$Tc in the North Pacific Ocean. Oceanographic parameters such as temperature, dissolved oxygen (DO), salinity and other basic properties of seawater were measured following routine GEOTRACES protocols.

**SI Table 1: Description of environmental water samples** including geographic coordinates of the sampling station, sample mass, salinity and Tc recovery during chemical sample preparation. (1) indicates that an additional $Fe(OH)_2$ co-precipitation was used for pre-concentration, (*) indicates that an average Tc recovery estimated from the subsequent samples is applied to the samples prepared before the $^{95m}$Tc spike was available (see main text for further details). n.a.: not available.

| Sample Name | Geographic Coordinates | Salinity (psu) | Sample weight (kg) | | Tc recovery (%) |
|---|---|---|---|---|---|
| Batch 1 | | | | | |
| BD-4-0m | 37.82 N, 143.88 E | n.a. | 11.209 ± 0.007 | | 89 ± 6* |
| BD-4-100m | | 34.691 | 11.200 ± 0.007 | | 89 ± 6* |
| BD-4-200m | | 34.492 | 11.195 ± 0.007 | | 89 ± 6* |
| BD-4-400m | | 34.276 | 9.352 ± 0.007 | | 89 ± 6* |
| BD-4-600m | | 33.999 | 11.456 ± 0.007 | | 77.9 ± 0.8 |
| BD-7-0m | 47.00 N, 160.08 E | n.a. | 8.021 ± 0.007 | | 84.5 ± 1.0 |
| BD-7-300m | | 34.003 | 10.156 ± 0.007 | | 94.8 ± 1.0 |
| BD-7-600m | | 34.250 | 10.970 ± 0.007 | | 95.5 ± 1.0 |
| BD-7-1000m | | 34.419 | 10.434 ± 0.007 | | 93.4 ± 1.0 |
| BD-7-2000m | | 34.607 | 10.401 ± 0.007 | | 89.6 ± 1.2 |
| BD-7-3000m | | 34.663 | 8.805 ± 0.007 | | 94.2 ± 0.8 |
| BD-7-5000m | | 34.685 | 10.635 ± 0.007 | | 90.6 ± 1.3 |
| Batch 2 | | | | | |
| BD-11-0m | 47.00 N, 180.00 E | n.a. | 10.898 ± 0.007 | | 33.6 ± 1.4 (1) |
| BD-11-200m | | 33.666 | 12.072 ± 0.007 | | 60.7 ± 3.1 (1) |
| BD-11-2000m | | 34.585 | 11.000 ± 0.007 | | 37.8 ± 1.6 (1) |
| BD-4-20m | 37.82 N, 143.88 E | 34.352 | 10.52 ± 0.01 | 5.309 ± 0.007 | 37.1 ± 1.9 (1) |
| | | | | 5.212 ± 0.007 | 45.2 ± 2.3 (1) |
| BD-15-600m | 50.83 N, 160.00 W | 34.229 | 10.100 ± 0.007 | 5.11 ± 0.01 | 91.1 ± 4.1 |
| | | | | 4.991 ± 0.007 | 79.2 ± 2.9 |
| Freshwater samples | | | | | |
| Antarctic snow | | n.a. | 10.299 ± 0.007 | | 91.8 ± 3.9 |
| Danube channel | 48.17 N, 16.48 E | n.a. | 8.676 ± 0.007 | | 88.9 ± 4.1 |
| Isar-1 (Ismaning) | 48.24 N, 11.67 E | n.a. | 9.163 ± 0.007 | | 83.1 ± 2.9 |
| Isar-2 (Garching) | 48.27 N, 11.68 E | n.a. | 8.462 ± 0.007 | | 33.5 ± 1.3 (1) |

## 1.2 Peat Bog Samples

The peat blocks from the Pürgschachen Moor (PSM, 47°34`53" N, 14°20`51" E, c. 630 m above sea level)) and Rotmoos (RM, 47°41`0" N, 15°9´18" E, 690 m above sea level), both located in Styria, Austria, were cut from the mire with a stainless-steel knife and stored in a freezer wrapped in PE foil. As the $^{99}$Tc concentration in the peat was unknown at the beginning of this study, these large volume samples were required to ensure the detection of a $^{99}$Tc global fallout signal at the cost of depth resolution. The sampling of the peat cores was carried out in the same way at both locations, which is described in more detail in (Hain, et al., in preparation) and were also stored in a freezer wrapped in PE foil. From the frozen peat bog blocks from the Pürgschachener Moor, slices of about 1 cm thickness, and four samples of 3 to 5 cm thickness (see SI Table 2) from the Rotmoos peat core were cut using a handsaw. In this first attempt to determine the $^{99}$Tc signal, no sophisticated protocols to maintain the chronology and avoid cross-contamination between the peat layers were applied. The samples were dried at 60°C for ~ 7 days and mechanically homogenized. Only one gram of this dried and homogenized peat was processed further for each AMS subsample of batch 2.

**SI Table 2: Description of peat bog samples** including sampling depth, sample mass of the frozen and dried peat and of the subsamples used for further processing and Tc recovery during chemical sample preparation (recovery determined with $^{95m}$Tc).

| Sample Name | Sampling | Sampling Depth (cm) | Frozen mass (g) | Dried mass (g) | Subsample mass ($g_{dry}$) | Tc recovery (%) |
|---|---|---|---|---|---|---|
| Batch 1 | | | | | | |
| RM75_10g | Block | 75 ± 5 | n.a. | 10.0 | 10.0 | 82.4 ± 0.9 |
| RM75_15g | Block | 75 ± 5 | n.a. | 15.3 | 15.3 | 89.4 ± 1.2 |
| Batch 2 | | | | | | |
| PSM5-15 | Block | 10 ± 5 | 43.45 | 3.951 | 1.00018 | 90.4 ± 3.5 |
| | | | | | 0.9939 | 41.9 ± 1.5 |
| PSM15-25 | Block | 20 ± 5 | 36.87 | 2.208 | 0.99241 | 93.2 ± 2.5 |
| | | | | | 1.00593 | 84.3 ± 3.4 |
| PSM25-35 | Block | 30 ± 5 | 30.15 | 3.920 | 1.00598 | 88.4 ± 3.6 |
| RM20-27 | Core | 23.8 ± 3.3 | 31.856 | 1.451 | 0.9796 | 90.0 ± 3.4 |
| RM43-52 | Core | 47.5 ± 4.5 | 32.138 | 3.415 | 1.0051 | 89.7 ± 1.7 |
| RM70-77 | Core | 73.8 ± 3.3 | 36.724 | 1.711 | 0.99740 | 96.3 ± 4.4 |

| | | | | | | |
|---|---|---|---|---|---|---|
| RM89-97 | Core | 93 ± 4 | 34.873 | 3.522 | 0.9917 | 99.4 ± 3.5 |

# 2 Sample preparation details

## 2.1 $^{95m}$Tc spike solution details

Within the scope of this work, the isotope $^{95m}$Tc ($t_{1/2}$ = (61.84 ± 0.07) d) [2] was produced as chemical yield tracer for sample preparation according to the procedure by Wacker et al. [3] using the reaction $^{95}$Mo(p,n)$^{95m}$Tc. A Molybdenum foil (0.757 g) was irradiated with 7.6 MeV protons at the Maier-Leibnitz-Laboratory in Munich. The proton energy was chosen well below 8 MeV to minimize co-production of $^{99}$Tc. The integrated particle flux over 54 h of irradiation was about 90 mC. The irradiated foil was dissolved in freshly opened $H_2O_2$ (30%, 35 mL). A portion (5 mL) of the resulting clear, yellow solution was removed and diluted to 35 mL total volume in a 40 mL poly propylene vial. This solution was loaded onto TEVA® column (0.3 g) pre-conditioned in 0.1 M $HNO_3$. The TEVA column was rinsed with 0.1 M $HNO_3$ (70 mL), followed by 1 M $HNO_3$ (70 mL) to elute the Mo fraction (clear yellow liquid). The resin must be white again prior to Tc-elution. $^{95m}$Tc was eluted from the column with 8 M $HNO_3$ (ultra grade, 35 mL).

## 2.2 Tc purification from water samples

The $^{95m}$Tc activity of each $^{95m}$Tc spike aliquot of 20 mL prepared for individual water samples was first measured with a high-purity Ge detector before it was added to the respective 10 L water sample and left to equilibrate for 24 h. For some of the 10 L water samples a $Fe(OH)_2$ co-precipitation was performed for pre-concentration, for others this step was skipped and LLX was directly performed on the 10 L sample. For the $Fe(OH)_2$ co-precipitation, the sample was neutralized with 30 mL 8 M NaOH and 10 mL of the homemade Fe-solution (containing 500 mg Fe) as well as $K_2S_2O_5$ (1 g/L seawater) were added. After waiting for 30 min the pH was slowly raised to ~8 using 8 M NaOH until the $Fe(OH)_2$ precipitate formed which was left to settle overnight. The next day the supernatant was discarded, and the remaining precipitate was dissolved by reducing the pH to 1 using 8 M $HNO_3$. At this point, 1-2 L of sample was left and LLX was performed in addition as preliminary test experiments showed an increased Ru suppression when LLX was used (see SI Figure 1). Tc was extracted two times with 5% TIOA-Xylene from which it was back-extracted with 0.5 M NaOH. The NaOH-phase was directly loaded onto the TEVA-column (Eichrom Industries, 2 mL, 200 mesh), which had been conditioned with 0.1 M $HNO_3$ beforehand. The column was rinsed with 0.1 M HCl, 0.1 M $HNO_3$ and 1 M $HNO_3$, respectively. Tc was finally eluted with 20 mL 8 M $HNO_3$. The Tc recovery was determined by measuring the $^{95m}$Tc activity in this solution by gamma spectrometry and calculating the ratio to the activity of the added spike determined in the same measurement geometry. Note that all these steps, but especially the handling of the organic solvents, must be carried out in a fume hood.

## 2.3. Investigations of Tc recovery and Ru suppression during purification of water samples

In contrast to other pre-concentration methods for Tc like co-precipitation with $Fe(OH)_2$, LLX offers an additional Ru suppression when extracting Tc from $HNO_3$ acidified media as in the case of the present samples. It should be noted that Ru can also be well extracted as $Ru(Cl)_3$ from media acidified with HCl. The suppression of Ru by LLX has been verified using 5 artificial seawater samples, each 5 L volume, prepared from sea-salt (Sigma Aldrich) with a concentration of 33 g/L.

These samples were spiked with circa 50 µg of Mo (ICP-Standard, $(NH_4)_6Mo_7O_{24}$ in $H_2O$ – 1000 mg/l, Merck), 500 ng of Ru ($RuCl_3$ in 7% HCl – 1000 mg/l, Merck), and 500 ng of $^{99}$Tc ((TCZ44, $NH_4TcO_4$ in $H_2O$, Eckert and Ziegler, Nuclitec.). Samples and a corresponding blank were prepared at the Karlsruhe Institute of Technology, Institute for Nuclear Waste Disposal (Germany) and the content of the three elements in the different fractions after each separation step was analysed by ICP-MS measurements (Thermo Element XR sector field ICP-MS, Thermo Fisher Scientific Inc., Waltham, USA). The results for the recoveries of the tracer isotopes of the three elements shown in SI Figure 1, clearly demonstrate that the main part of the Ru suppression already happens during LLX extraction with a decontamination factor of about $10^{-3}$ (SI Figure 1a). With the TEVA resin an additional Ru suppression of only a factor 5 (Figure 1b) could be achieved which might be due to the rather strong alkaline column loading solution [4]. Due to the very good instrumental Ru suppression by the GAMS setup, we decided to optimize the turnaround time of chemical sample preparation by skipping an additional neutralization step before the TEVA column. Mo, however, could only be removed using TEVA-resin.

Typical extraction efficiency of Tc from the artificial sea water samples acidified with $HNO_3$ to pH 1 was about 95% (SI Figure 1a). However, due to bubble formation in the TEVA resin, the elution of Tc was disturbed, as reflected by the lower chemical yield for samples 3 & 5 compared to the other samples for which a yield of >86% was obtained.

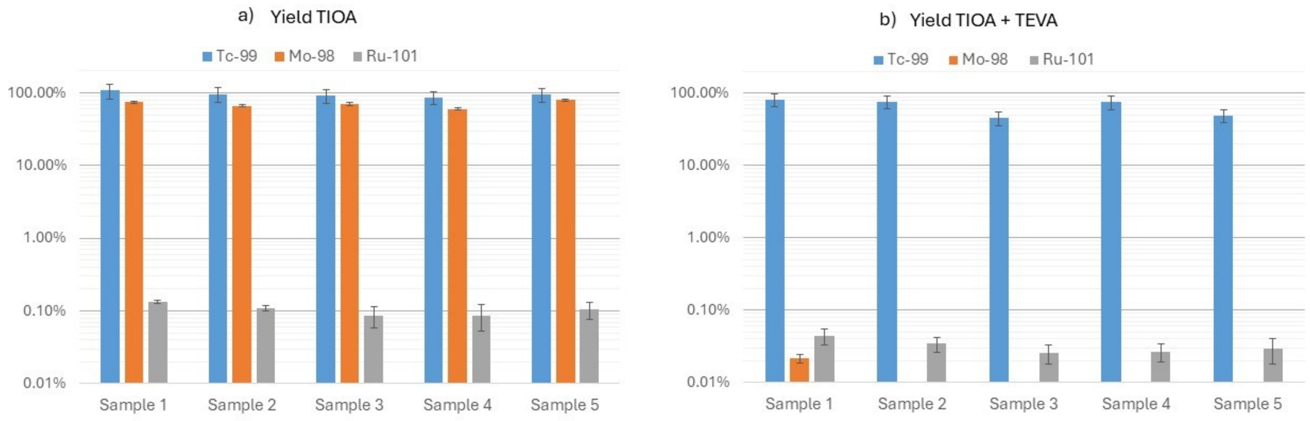


**SI Figure 1: Yield of tracers (Tc, Mo and Ru) for LLX with 5% TIOA-Xylene (a) and the overall chemical recovery of tracers after the pre-concentration and separation step (b).** $^{98}$Mo concentrations are not shown in (b) apart for sample 1 as they were below the detection limit, i.e. decontamination factor > $10^{-4}$.

## 2.4 Tc purification from peat samples

The samples were dried in beakers at 60°C in the drying oven for ~ 7 days and then mechanically homogenized. The $^{95m}$Tc spike in 5 ml $HNO_3$ conc. and additional 5 mL $HNO_3$ conc. were added to the dried peat in PTFE vessels for microwave digestion as described in [5]. After the microwave digestion the sample was evaporated to 1 mL at 200°C and diluted to 100 ml with Milli-Q water. LLX was performed with 2 × 30 mL 5% TIOA-Xylene and back-extracted with 2 × 20 mL of 0.5 M NaOH. The NaOH-Phase was directly loaded onto the TEVA-column (Eichrom Industries, 2 mL, 200 mesh), which had been conditioned with 0.1 M $HNO_3$ beforehand. The column was rinsed with 10 mL 0.1 M HCl, 40 ml 0.1 M $HNO_3$ and 40 ml 1 M $HNO_3$, respectively. Tc was finally eluted with 20 mL of 8 M $HNO_3$. The sample was evaporated to 5 mL and the Tc-recovery was measured on the high purity Ge-Detector.

### 2.5 Investigations of Tc recovery during evaporation

The recovery of Tc during evaporation in 8 M $HNO_3$ was investigated at 80°C and 200°C. Around 0.3 Bq of $^{95m}Tc$ was added to 10 mL 8 M $HNO_3$, and analysed in our standard vial with the high purity Ge-detector evaluating the emitted gamma radiation at 204.3 keV. After each evaporation step, the sample was diluted in 10 mL 8 M $HNO_3$, to maintain the same geometry, allowing direct comparison of the count rate to the reference measurement before the evaporation step. Within the uncertainties no Tc-losses were observed.

**SI Table 3: $^{95}Tc$ recovery in 8M $HNO_3$ after evaporation to 1 mL and to dryness at 80°C and 200°C indicating no major losses for both temperatures.** The uncertainty of the final recovery is given by uncertainty propagation of counting statistics of two measurements, and the uncertainty of the background correction. The background in the range of 203-206 keV is (3.06 ± 0.35) $\times 10^{-4}$ counts/s.

| **Evaporation step** | **Recovery [%]** |
|---|---|
| evaporated to 1 mL at 80°C | 97 ± 7 |
| evaporated to dryness at 80°C | 99 ± 4 |
| evaporated to 1 mL at 200°C | 102 ± 6 |
| evaporated to dryness at 200°C | 98 ± 8 |

## 3 Data evaluation for the GAMS setup at the MLL

### 3.1 Background discrimination with the gas-filled magnet in combination with ToF

The ToF telescope in front of the GAMS was equipped with a start and a stop channel-plate detector with a flight path of 3.5 m. The time resolution of the ToF telescope achieved in these measurements was about 0.5% (FWHM). The time signal is furthermore correlated to the energy loss signals of the ion in the ionisation chamber, leading to an unambiguous determination of the ion mass. Neighbouring, highly abundant Mo and Ru isotopes with different masses are therefore clearly separated in the ToF spectrum (see Figure SI 2a), left), whereas the remaining stable isobar $^{99}Ru$ can be identified by its differential energy loss in the ionisation chamber (Figure SI 2b) middle and right in comparison to 2a) right). In order to identify the $^{99}Tc^{12+}$ counts, narrow software cuts were applied on the initial ToF, the ΔE1-ΔE4 energy loss and the total energy ($E_{tot}$) signal (see SI Figure 2b) using standard samples, for which the $^{99}Tc$ count rate was significantly higher than that from $^{99}Ru$, and blank samples for which the mass-99 signals were due to $^{99}Ru$ only. Finally, a two-dimensional region of interest (ROI) was defined in the ΔE5 vs position diagram (see Figure SI 2b, right). The unidentified species marked by the dashed, green circle which is also located close to the $^{99}Ru$ signal in the ΔE5 vs position spectrum on the right, can be easily distinguished by the other energy loss signals (not shown), whereas the species marked by the red, solid circle, identified as $^{97}Mo^{12+}$ closely follows the $99^{12+}$ signal on all anodes of the ionisation chamber. Only a narrow window on the ToF signal (lower row, left), removes this species (compare upper row, right with lower row, middle), leading to a sufficient background suppression in the ROI for $^{99}Tc^{12+}$ in combination with windows on the other 8 signals of the ionisation chamber for the ΔE5 vs position spectrum (lower row, right). In the described configuration including ToF, it

has proven to be advantageous for the separation of $^{99}Ru$ and $^{99}Tc$ to decrease the pressure in the ionisation chamber from 32.5 mbar [5] to 25.5 mbar.

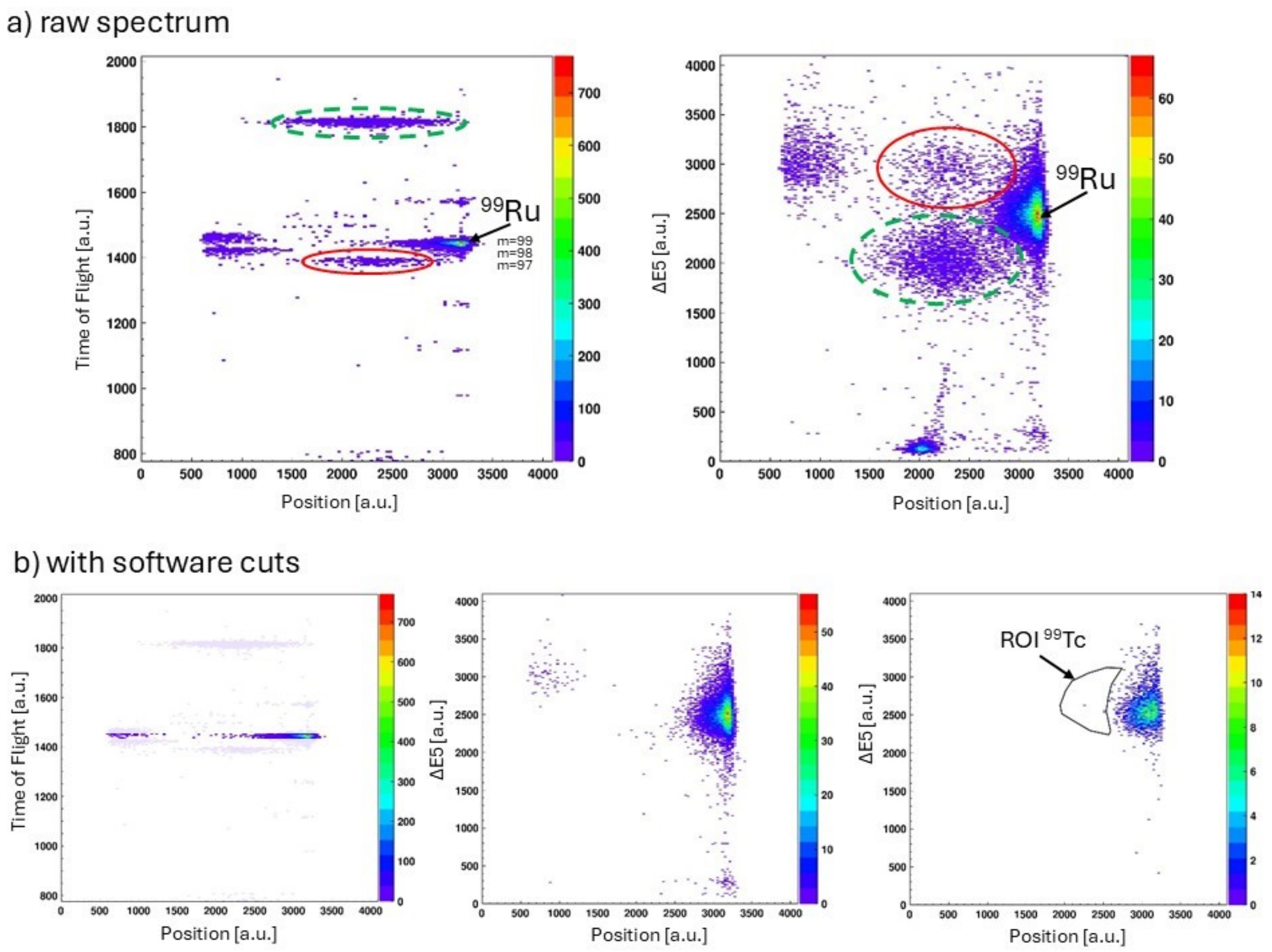


**SI Figure 2: ToF vs position (left) and ΔE5 vs position diagram (middle, right) spectra with the settings of the AMS setup on mass 99 obtained for a process blank without (a) and with software cuts to select $^{99}Tc$ (b).** ΔE5 denotes the last anode (E5) of the ionisation chamber. A software cut on the TOF signal for m=99 (see 2b left) removes the unknown species marked by the solid red and dashed green lines in Fig. 2a) which led to background in the region of the ΔE5 vs position diagram (Fig. 2a right) where the $^{99}Tc$ events are expected (compare Fig. 2b middle). Application of software cuts on the ΔE1-ΔE4 energy loss signals leads to further reduction of especially isobaric background in the final ΔE5 vs position diagram (Fig. 2b right) in which a region of interest (ROI, solid black line) was defined to select the events identified as $^{99}Tc$.

### 3.2 $^{99}$Tc quantification

The narrow software cuts led to a rather low acceptance of only about 1% of the $^{99}Tc^{12+}$ counts with respect to the last beam current measurement in front of the ToF path, considerably decreasing the overall detection efficiency. The acceptance is taken into account when calculating the number of $^{99}$Tc atoms in the sample by introducing a corresponding normalisation factor (τ) (= nominal ratio/measured ratio) which is determined by measuring standard samples spiked with about $10^{10}$ atoms of $^{99}$Tc and a known $^{99}$Tc/$^{93}$Nb atom ratio. Standard samples in comparison to the process blanks were also used to define the ranges for the software cuts on the different parameters as well as the ROI. Process blanks, prepared from high-purity reagents, however, do not represent the variable Ru content of the environmental samples sufficiently well to do a simple blank correction for the final result. A background correction for $^{99}$Ru events leaking into the region of interest was therefore applied which was assumed to be proportional to the number of counts recorded in the ROI for $^{99}$Ru (*ev(Ru)*). To estimate this background, the Ru fraction scattered into the $^{99}$Tc ROI ($ev(Ru)_{ROI\text{-}Tc}$) was estimated from measuring a sample with high $^{99}$Ru content ("Ru standard" spiked with about ~2.4 × $10^{16}$ atoms). A machine blank made from pure $Nb_2O_5$ providing about a factor 50 lower Ru count rates, was used to monitor potential drifts during the runtime of the measurement campaign without risking increasing the Ru level in the ion source significantly. This Ru correction factor $f_{Ru}$ with

$$f_{Ru=}\, ev(Ru)_{ROI\,-\,Tc}/ev(Ru) \tag{1}$$

allows calculation of an average number of misidentified background events in the Tc ROI depending on the Ru content of the sample which were then subtracted from the Tc events for all sample types (blanks, standards, environmental samples). Both, the normalisation factor as well as the Ru correction factor, were determined at least once for each tuning of the AMS setup. However, the latter turned out to be a critical parameter for the evaluation which needs to be monitored more closely, especially if the Ru count rate is not suppressed as in the case of the GAMS.

Normalising the obtained events ev(Tc) to the $Nb^{11+}$ current $I_{Nb\text{-}C1'}$ measured in the Faraday cup in front of the ToF and taking the correction factors τ and $f_{Ru}$ into account gives the number of $^{99}$Tc atoms N(Tc) in the AMS target:

$$N(Tc) = N(Nb) \cdot \tau \cdot \frac{(ev(Tc) - ev(Ru) \cdot f_{Ru})}{I_{Nb\,-\,C1'} \cdot t/q \cdot e} \tag{2}$$

with $N(Nb)$ being the number of Nb atoms in the target, t being the measurement time, and q denoting the selected charge state (here: 11+). This result was corrected by the chemical yield and divided by the sample mass to obtain the $^{99}$Tc concentration in the initial sample. The corresponding process blank level has been obtained in the same way. Results from individual measurement runs or beam times have been averaged by calculating the weighted mean value.

## 4 Data evaluation at HIAF

### 4.1 Background discrimination with the 8-anode gas ionisation chamber

An eight-anode ionisation chamber [3] with a 5 × 5 $mm^2$, 50 nm thick SiN entrance window, filled with isobutane gas is used for the final detection of $^{99}Tc$. The total energy ($E_{Tot}$) as well as the energy loss signals from each of the eight anodes of the detector ($\Delta E_1$-$\Delta E_8$) were recorded for each event in the detector. Owing to the differences in the stopping power (differential energy loss) of the $^{99}Tc$ and its stable isobar $^{99}Ru$ the individual energy loss signals and sums of them, allow for the separation and identification of $^{99}Tc$ from its isobar $^{99}Ru$. The detector gas pressure was set so that only about 5% of $^{99}Ru$ reach the last anode. In contrast, ~65% of the $^{99}Tc$ ions reached the last anode under these conditions. The total energy information allowed to discriminate against potential interferences from particles in different charge states but with a similar mass over charge ratio to $^{99}Tc^{13+}$. Software cuts on the $^{99}Tc$ peaks in the $\Delta E_2$, $\Delta E_6$, $\Delta E_7$, $\Delta E_8$, and $E_{Tot}$ spectra were defined using standard samples for which the $^{99}Tc$ count rate was significantly higher than that from $^{99}Ru$, and blank samples for which the mass-99 signals were due to $^{99}Ru$ only. A 2-dimensional spectrum of $E_{12345}$ (sum of signals $\Delta E_1$-$\Delta E_5$) vs $E_{678}$ of events that fell within all five of these cuts was then created, and a region of interest was defined that accepted as many of the $^{99}Tc$ events as possible while rejecting almost all the $^{99}Ru$ events. The corresponding ROI for $^{99}Ru$ was determined analogously using "Ru standards" spiked with $10^{10}$-$10^{11}$ atoms per sample. Much lower Ru concentrations were used than for the GAMS measurements, due to the higher risk for cross-contamination in the Multi-Cathode ion source of the HIAF compared to the Single-Cathode ion source of the MLL and to avoid unnecessarily high count rates in

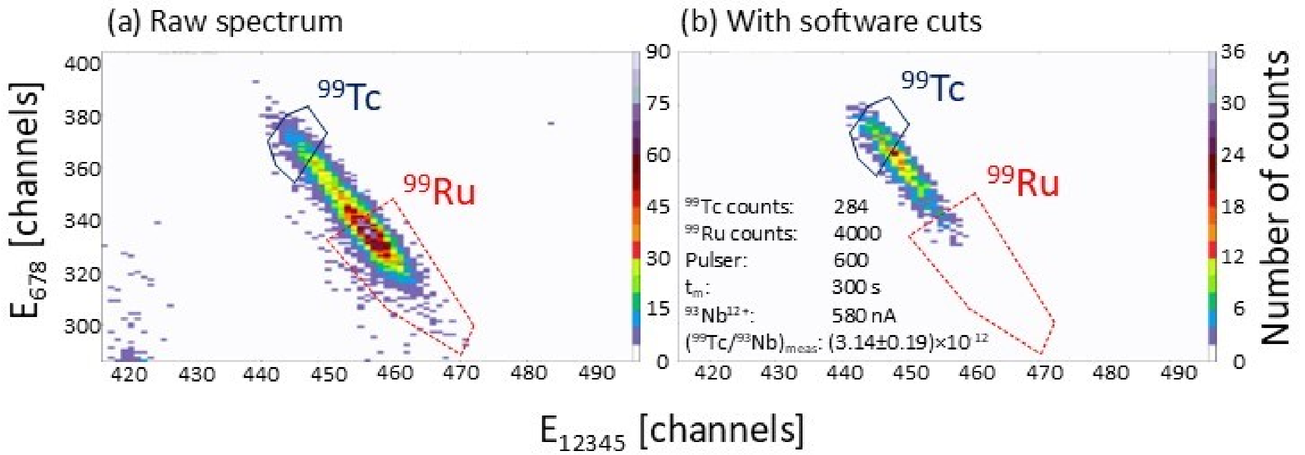


the ionisation chamber. Representative 2-dimensional spectra with the final $^{99}Tc$ ROI for an environmental sample are shown in SI Figure 3.

**SI Figure 3: Sum of the energy loss signals on anode 1-5 vs anode 6-8 spectra with the settings of the AMS setup on mass 99 obtained for environmental sample RM20-27.** (a) Raw spectrum without any software cuts applied to select $^{99}Tc$ (b) spectrum with all software cuts applied. The ROIs derived for $^{99}Tc$ (upper left) and $^{99}Ru$ (lower right) are marked in both graphs by the blue solid line and the red dashed line, respectively.

### 4.2 Reproducibility of the correction factors

Depending on the tuning, the $^{99}Ru$ induced counts in the $^{99}Tc$ ROI (defined as the Ru correction factor $f_{Ru}$ given by equation (1)) ranged from 0.02% to 0.04% for the Ru standards, with a relative uncertainty of around 14-50 % (compare SI table 4). Even though the relative uncertainty of $f_{Ru}$ is rather large, the uncertainty for the corrected $^{99}Tc$ events is dominated by counting statistics on Tc. Depending on the tuning, the average normalisation factor varied from 19.6 ± 3.8 to 27 ± 6 with

relative uncertainties ranging from 10-20%. As this relative uncertainty is added quadratically to the relative uncertainty of the $^{99}Tc$ events, it is a significant contribution to the final uncertainty of the $^{99}Tc/^{93}Nb$ ratio from an individual run.

**SI Table 4: Tuning dependent Ru correction factor $f_{Ru}$ measured on Ru standards and normalisation factor $\tau$ measured on corresponding Tc standards** for the six different tunings, with the first four tunings used for the water samples and tunings 5 and 6 used for the measurement of the peat samples.

| tuning | # runs on Ru standards | average Ru-correction factor $f_{Ru}$ | # runs on Tc standards | average normalisation factor $\tau$ |
|---|---|---|---|---|
| 1 | 2 | $(4.4 \pm 1.3) \times 10^{-4}$ | 2 | 19.6 ± 3.8 |
| 2 | 2 | $(2.6 \pm 1.2) \times 10^{-4}$ | 5 | 24.5 ± 4.3 |
| 3 | 3 | $(2.9 \pm 0.7) \times 10^{-4}$ | 4 | 18.4 ± 2.1 |
| 4 | 4 | $(2.9 \pm 0.7) \times 10^{-4}$ | 6 | 22.1 ± 4.3 |
| 5 | 3 | $(3.8 \pm 0.5) \times 10^{-4}$ | 4 | 17.8 ± 1.5 |
| 6 | 3 | $(2.13 \pm 0.42) \times 10^{-4}$ | 14 | 27 ± 6 |

### 4.3 Reproducibility of the external standards

Figure 4 demonstrates the reproducibility of the measurement results by showing the determined $^{99}Tc$ content in the respective AMS sample of the three individual water standards for subsequent runs. The $^{99}Tc/^{93}Nb$ ratios have been normalised to the nominal value of standard #1 of $(1.22 \pm 0.04)\times10^9$ at/sample, and, considering the other two standards as unknowns, the $^{99}Tc$ content in the sputter target have been calculated. Although one (#3) of the three standards has not undergone chemical purification, no systematic difference in the results is observed and therefore all values were included in the evaluation of the reproducibility. The same approach was used for the three standards corresponding to the measurement of the peat bog samples. Results for the $^{99}Tc^{13+}$ count rate in comparison to the measured $^{93}Nb^{12+}$ current for the individual runs are listed in SI Table 5.

The scatter of the measured $^{99}Tc$ concentration values of the standards is used as an indicator of the reproducibility. The averaged measured concentration were $(1.25 \pm 0.21)\times10^9$ and $(1.24 \pm 0.20)\times10^9$ atoms per sample for the water sample standards and the peat sample standards, respectively. Thus, the overall reproducibility of the normalisation with respect to the $^{93}Nb^{12+}$ current, was 16% in both cases. This represents a significant improvement compared to 30% - 46% achieved for the normalisation to $^{93}Nb$ at the MLL [6,7]. Most importantly, we do not observe a systematic deviation over time which would point to a fractionation by differential sputtering of Nb and Tc. The total measurement time, especially of standard 3 (2400 s), corresponds well to the average measurement time for the environmental samples and therefore shows the robustness of the non-isotopic normalisation within the determined uncertainty of 16%. Experiments at HIAF indicated this higher reproducibility can be achieved by allowing the sputtering process in the ion source to reach equilibrium

conditions. Consequently, all samples in the subsequent measurements at HIAF including the unknown environmental samples were sputtered for about 10-15 minutes before the actual measurement was started.

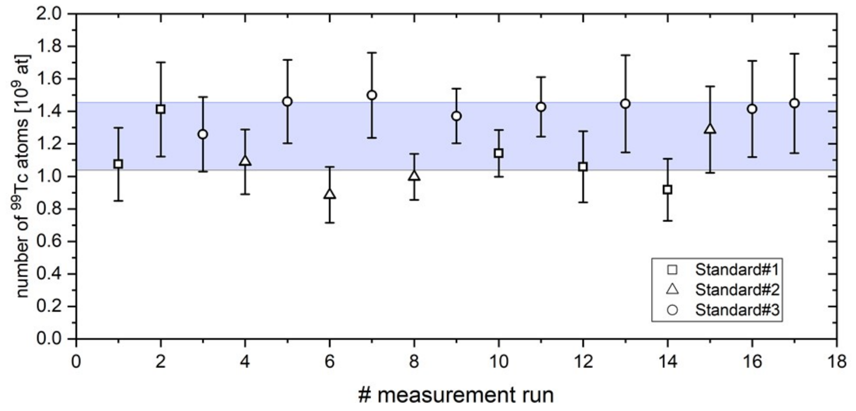


**Fig. 4:** $^{99}$Tc-content per standard sample for each measurement run, normalised to the nominal concentration of (1.22 ± 0.04)×$10^9$ at/sample of standard #1. The symbols refer to the individual samples, and the blue band represents the 1 sigma standard deviation of all standards.

**SI Table 5: Data from standard samples from individual measurement runs at the HIAF to determine reproducibility**: Recorded Nb currents in comparison to $^{99}$Tc count rates.

| Name | run | counting time per run [s] | total collected $^{99}$Tc counts | $^{99}$Tc count rate [1/s ×100] | average $^{93}Nb^{12+}$-current [nA] | Ru-corrected ratio $^{99}$Tc /$^{93}$Nb [×$10^{-14}$] |
|---|---|---|---|---|---|---|
| **Tc and Ru standards for water sample measurement** | | | | | | |
| Tc Standard #1 | 1 | 300 | 147 ± 12 | 0.490 ± 0.041 | 7.5 | 251 ± 21 |
| | 2 | 300 | 191 ± 14 | 0.638 ± 0.047 | 7.4 | 330 ± 24 |
| | 3 | 296 | 241 ± 16 | 0.81 ± 0.05 | 11.0 | 284 ± 18 |
| | 4 | 299 | 229 ± 16 | 0.77 ± 0.05 | 13.5 | 219 ± 15 |
| | 5 | 300 | 212 ± 15 | 0.707 ± 0.049 | 14.3 | 190 ± 13 |
| Tc Standard #2 | 1 | 299 | 137 ± 12 | 0.459 ± 0.039 | 7.5 | 235 ± 20 |
| | 2 | 299 | 86 ± 9 | 0.286 ± 0.031 | 5.7 | 191 ± 21 |
| | 3 | 300 | 118 ± 11 | 0.394 ± 0.036 | 5.3 | 287 ± 26 |
| | 4 | 300 | 233 ± 15 | 0.78 ± 0.05 | 9.7 | 308 ± 20 |
| Tc Standard #3 | 1 | 300 | 139 ± 12 | 0.462 ± 0.039 | 6.9 | 259 ± 22 |
| | 2 | 300 | 208 ± 14 | 0.692 ± 0.048 | 8.8 | 301 ± 21 |
| | 3 | 300 | 223 ± 15 | 0.74 ± 0.05 | 9.2 | 309 ± 21 |
| | 4 | 300 | 297 ± 17 | 0.99 ± 0.06 | 10.1 | 377 ± 22 |
| | 5 | 300 | 205 ± 14 | 0.685 ± 0.048 | 6.7 | 393 ± 27 |
| | 6 | 300 | 223 ± 15 | 0.743 ± 0.05 | 8.6 | 331 ± 22 |

| | | | | | | |
|---|---|---|---|---|---|---|
| | 7 | 300 | 184 ± 14 | 0.612 ± 0.045 | 7.3 | 323 ± 24 |
| | 8 | 300 | 162 ± 13 | 0.540 ± 0.042 | 6.3 | 331 ± 26 |

### 4.4 Collected raw data

Typical transmissions from the Faraday cup after the injection magnet to the cup directly in front of the detector at HIAF were 4-5%. Counting times of the radionuclide were 100-600 s, and these were sandwiched between 10 s $^{93}Nb^{12+}$ current measurements (see SI Table 6 for an overview of the collected raw data).

**SI Table 6: Collected AMS raw data of all samples from individual cathodes measured at the HIAF**: Recorded Nb currents, events detected in the $^{99}Tc$ and $^{99}Ru$ region of interest, and the calculated $^{99}Tc/^{93}Nb$- ratio.

| Name | #runs | Average counting time per run [s] | total collected $^{99}Tc$ counts | total collected $^{99}Ru$ counts [×10$^{4}$] | average $^{99}Tc$ count rate [1/s ×100] | average $^{99}Ru$ count rate [1/s] | $^{93}Nb^{12+}$-current [nA] | Ru-corrected ratio $^{99}Tc/^{93}Nb$ [×10$^{-14}$] |
|---|---|---|---|---|---|---|---|---|
| **Tc and Ru standards for water sample measurement** | | | | | | | | |
| Tc Standard #1 | 5 | 299 | 1033 | 4.0 | 69 ± 6 | 26.7 ± 3.9 | 10.7 ± 1.4 | 255 ± 24 |
| Tc Standard #2 | 4 | 300 | 577 | 1.3 | 48 ± 11 | 10.5 ± 2.1 | 7.1 ± 1.0 | 255 ± 26 |
| Tc Standard #3 | 8 | 300 | 1643 | 1.3 | 69 ± 6 | 5.3 ± 0.4 | 8.0 ± 0.5 | 328 ± 15 |
| Ru standard #1 | 4 | 1036 | 171 | 66 | 4.0 ± 0.4 | 149 ± 28 | | |
| Ru standard #2 | 7 | 1259 | 279 | 83 | 6.9 ± 2.0 | 200 ± 50 | | |
| **Water chemistry blanks** | | | | | | | | |
| Blank-water #1 (9E12 Ru) | 3 | 599 | 10 | 3.4 | 0.56 ± 0.18 | 19 ± 7 | 10.2 ± 2.7 | < 0.8 |
| Blank-water #2 (9E13 Ru) | 2 | 599 | 14 | 5.1 | 1.2 ± 0.7 | 42 ± 14 | 9.8 ± 1.8 | < 1.5 |
| **Environmental water samples** | | | | | | | | |
| Antarctic | 4 | 584 | 19 | 7.4 | 0.83 ± 0.40 | 32 ± 7 | 12.3 ± 1.7 | < 0.7 |
| BD-11-2000m-A | 4 | 600 | 9 | 2.6 | 0.38 ± 0.16 | 10.8 ± 0.7 | 9.2 ± 0.7 | < 0.9 |
| BD-11-2000m-B | 4 | 624 | 8 | 2.9 | 0.33 ± 0.12 | 11.5 ± 1.0 | 11.7 ± 1.3 | < 0.6 |
| BD-15-600m-1/2 | 5 | 600 | 45 | 6.8 | 1.50 ± 0.33 | 23 ± 5 | 11.3 ± 1.2 | 2.6 ± 1.0 |
| BD-15-600m-2/2 | 2 | 583 | 7 | 1.3 | 0.60 ± 0.23 | 11 ± 6 | 7.5 ± 3.0 | 2.1 ± 1.5 |
| BD-11-200m-B | 6 | 598 | 69 | 5.8 | 1.93 ± 0.31 | 16 ± 4 | 8.0 ± 0.6 | 7.0 ± 1.2 |
| BD-04-20m-1/2 | 4 | 599 | 55 | 4.8 | 2.3 ± 0.5 | 19.9 ± 3.0 | 10.9 ± 1.2 | 5.9 ± 1.3 |
| BD-04-20m-2/2 | 4 | 599 | 77 | 8.6 | 3.2 ± 0.6 | 36 ± 6 | 10.8 ± 1.8 | 8.0 ± 1.9 |

| | | | | | | | | |
|---|---|---|---|---|---|---|---|---|
| BD-11-0m-A | 6 | 587 | 56 | 5.7 | 1.58 ± 0.30 | 16.3 ± 2.8 | 8.7 ± 1.0 | 4.7 ± 1.0 |
| BD-11-0m-B | 4 | 593 | 44 | 4.6 | 1.86 ± 0.34 | 19.2 ± 1.8 | 12.9 ± 2.0 | 3.8 ± 0.9 |
| Isar-1 (Ismaning) | 4 | 600 | 503 | 5.0 | 21 ± 5 | 21 ± 5 | 10.9 ± 2.4 | 70.2 ± 3.6 |
| Isar-2 (FZ-Garching) | 6 | 597 | 188 | 19 | 5.3 ± 0.6 | 52 ± 4 | 4.10 ± 0.22 | 33.7 ± 6.1 |
| Danube Channel | 3 | 600 | 446 | 3.7 | 25 ± 5 | 20.6 ± 3.8 | 8.8 ± 1.0 | 104 ± 11 |
| **Tc and Ru standards for peat sample measurement** | | | | | | | | |
| Tc Standard #4 | 8 | 300 | 1760 | 3.7 | 73 ± 11 | 15.2 ± 2.6 | 10.6 ± 1.8 | 279 ± 24 |
| Tc Standard #5 | 6 | 299 | 1404 | 3.1 | 78 ± 10 | 17.4 ± 2.1 | 12.2 ± 1.6 | 247 ± 9 |
| Tc Standard #6 | 4 | 299 | 785 | 0.77 | 66 ± 15 | 6.4 ± 1.4 | 11.5 ± 1.8 | 212 ± 22 |
| Ru standards #3 | 5 | 538 | 191 | 68 | 7.3 ± 1.3 | 270 ± 60 | | |
| Ru standards #4 | 1 | 293 | 97 | 27 | 33.1 ± 3.4 | 918 ± 2 | | |
| **Peat chemistry blanks** | | | | | | | | |
| Blank-peat #1 (not spiked) | 1 | 600 | 3 | 0.21 | 0.50 ± 0.29 | 3.53 ± 0.08 | 3.1 ± 0 | 5.3 ± 3.6 |
| Blank-water #2 (9E13 Ru) | 2 | 599 | 32 | 8.6 | 2.7 ± 0.8 | 71 ± 12 | 13.6 ± 2.2 | 3.1 ± 1.5 |
| **Environmental peat samples** | | | | | | | | |
| PM5-15_1 | 4 | 444 | 1114 | 1.5 | 67 ± 9 | 10.0 ± 2.4 | 8.2 ± 2.0 | 343 ± 47 |
| PM5-15_1 | 3 | 400 | 385 | 3.3 | 37 ± 10 | 33 ± 11 | 11.7 ± 3.6 | 125 ± 9 |
| PM15-25_1 | 4 | 449 | 972 | 5.0 | 66 ± 21 | 29 ± 8 | 14 ± 5 | 212 ± 24 |
| PM15-25_2 | 2 | 295 | 280 | 0.81 | 48 ± 8 | 13.7 ± 3.9 | 13.8 ± 2.7 | 133 ± 8 |
| PM25-35 | 5 | 581 | 163 | 4.1 | 5.5 ± 1.5 | 13.8 ± 3.6 | 11.8 ± 3.2 | 16.4 ± 3.6 |
| RM20-27 | 4 | 421 | 1658 | 2.0 | 99 ± 16 | 12.5 ± 2.0 | 11.3 ± 2.0 | 341 ± 34 |
| RM43-52 | 5 | 596 | 68 | 4.2 | 2.3 ± 0.7 | 14.0 ± 3.3 | 12.6 ± 3.0 | 6.3 ± 1.6 |
| RM70-77 | 5 | 599 | 19 | 2.7 | 0.63 ± 0.19 | 8.9 ± 2.3 | 6.1 ± 1.2 | 2.5 ± 1.0 |
| RM89-97 | 5 | 596 | 82 | 5.4 | 2.8 ± 0.4 | 18.2 ± 3.4 | 15.1 ± 3.3 | 6.8 ± 1.4 |

## 5 Details on the estimation of the global fallout $^{99}$Tc inventory

From the two depth distributions obtained from sample batch 2, the Pacific Ocean water samples and the peat samples, a global inventory of $^{99}$Tc from global fallout can be estimated. For the water samples, assuming a linear change between data points and integrating the depth profile yields an areal concentration of $(6.6 \pm 1.6)\times10^{12}$ atoms of $^{99}$Tc per m². The background level was defined by the deepest samples of the profile corresponding to the lowest $^{99}$Tc concentration. Global fallout was not distributed homogeneously and shows significant variation with latitude [8]. Hence, calculation of

the global fallout inventory from areal concentrations is scaled according to latitude using the measured $^{90}$Sr deposition data [9]. The samples of the present work were collected within the 40–50°N latitudinal band, which received 17% of the global fallout, despite covering only 6% of the Earth's surface. After correcting for background, scaling with the $^{90}$Sr data and extrapolating to the Earth's surface area of $5.1\times10^{8}$ km$^2$, the global inventory is calculated as $(1.2 \pm 0.5)\times10^{27}$ atoms $^{99}$Tc corresponding to $(120 \pm 50)$ TBq. Converting weight concentration to volume concentration for peat samples, requires accurate information about the sample volume. This was available for the RM peat core samples, which were collected using a cutting tool with a defined circular area of 6 cm in diameter and stored in tubes, preserving the original depth measurements. For the four RM samples, the average density was determined to be $(0.025 \pm 0.004)$ g dry/cm$^3$. This density was used for the three PM samples to combine the datapoints for this estimation. As no measurement data is available for the surface of the peat bog, we distinguish between two limiting scenarios: 1) The $^{99}$Tc concentration decreases to background levels at the surface or 2) the surface concentration stays constant at the value of the closest data point (PSM5-15cm). This yields areal concentrations of $(7.2 \pm 2.5)\times10^{12}$ atoms/m² and $(9.0 \pm 2.7)\times10^{12}$ atoms/m² for scenario 1) and 2), respectively. Following the same approach as for the water samples, i.e. background correction, and scaling with $^{90}$Sr deposition data, we obtain for scenario 1) an estimate for the global inventory of $(1.5 \pm 0.5)\times10^{27}$ atoms $^{99}$Tc ((150 ± 50) TBq) and for scenario 2) $(1.8 \pm 0.6)\times10^{27}$ atoms $^{99}$Tc (or (190 ± 60) TBq).

## References


1. Hain K, Faestermann T, Fimiani L, et al. Plutonium Isotopes ($^{239–241}$ Pu) Dissolved in Pacific Ocean Waters Detected by Accelerator Mass Spectrometry: No Effects of the Fukushima Accident Observed. *Environ Sci Technol* 2017; 51: 2031–2037.

2. NuDat database, https://www.nndc.bnl.gov/nudat/ (accessed 8 January 2026).

3. Wacker L, Fifield LK, Tims SG. Developments in AMS of 99Tc. *Nuclear Instruments and Methods in Physics Research Section B: Beam Interactions with Materials and Atoms* 2004; 223–224: 185–189.

4. Uchida S, Tagami K. Separation and concentration of technetium using a Tc-selective extraction chromatographic resin. *J Radioanal Nucl Chem* 1997; 221: 35–39.

5. Quinto F, Busser C, Faestermann T, et al. Ultratrace Determination of $^{99}$Tc in Small Natural Water Samples by Accelerator Mass Spectrometry with the Gas-Filled Analyzing Magnet System. *Anal Chem* 2019; 91: 4585–4591.

6. Koll D, Busser C, Faestermann T, et al. Recent developments for AMS at the Munich tandem accelerator. *Nuclear Instruments and Methods in Physics Research Section B: Beam Interactions with Materials and Atoms* 2019; 438: 180–183.

7. Quinto F, Busser C, Faestermann T, et al. Ultratrace Determination of$^{99}$ Tc in Small Natural Water Samples by Accelerator Mass Spectrometry with the Gas-Filled Analyzing Magnet System. *Anal Chem* 2019; 91: 4585–4591.

8. Machta L, List RJ. Discussion of Meterological Factors and Fallout Distribution. In: *Environmental Contamination from Weapon Tests*. Oak Ridge, Tennessee: U.S. Atomic Energy Commission, 1958, pp. 327–338.

9. UNSCEAR. *Sources and Effects of Ionizing Radiation - Annex C*. I, New York: UNSCEAR, January 2000.
